# ASAS-NANP SYMPOSIUM: MATHEMATICAL MODELING IN ANIMAL NUTRITION: quantum computing in agricultural sciences: from theory to reality

**DOI:** 10.1093/jas/skaf445

**Published:** 2025-12-18

**Authors:** Luis O Tedeschi

**Affiliations:** Department of Animal Science, Texas A&M University, College Station, TX, 77843-2471

**Keywords:** agriculture technology, hybrid intelligence, modeling, precision livestock farming, quantum computing, quantum simulation

## Abstract

Quantum computing (QC) represents a revolutionary paradigm in information processing, leveraging quantum mechanical phenomena (superposition, entanglement, quantum interference, and quantum tunneling) to perform calculations in fundamentally different ways than classical computing (CC). While CC processes information sequentially through Boolean logic operations on discrete binary states (0 s and 1 s), quantum computers manipulate qubits that can exist in superpositions of states, enabling parallel operations on exponentially large state spaces. Despite claims regarding “quantum supremacy,” QC remains in its early developmental stages, comparable to the CC of the 1950s and 1960s. True quantum supremacy, where quantum computers demonstrate definitive, practical advantages over classical computers for well-defined tasks, has not yet been established. Practical applications face real challenges, i.e., decoherence, high error rates, and demanding error correction requirements. Three developmental phases are projected: noisy intermediate-scale quantum systems by 2030, broad quantum advantage from 2030 to 2040, and full-scale fault tolerance after 2040. Does QC offer solutions to fundamental problems that classical systems, including supercomputers and artificial intelligence, cannot already resolve? While conventional technologies continue to advance agricultural capabilities through machine learning (ML) and complex optimization, quantum approaches may potentially transform domains that require molecular-level simulations (such as soil chemistry and rumen microbial interactions) or exponentially complex optimization problems in resource allocation. Quantum ML models, such as quantum neural networks, generative adversarial networks, and autoencoders, are being explored in quantum–classical hybrids, which have shown potential for faster optimization and higher-dimensional data representation; but, these advantages remain largely conceptual. The value proposition of QC in agriculture ultimately depends on whether the field’s most pressing challenges involve quantum mechanical processes that classical computers cannot simulate efficiently or optimization problems of such complexity that quantum algorithms would provide substantial practical advantages over classical approaches. The agricultural community must also address societal implications, such as access equity, data ownership, algorithmic transparency, and educational preparedness for this emerging technology.

## Teaser

Quantum computing might simulate rumen fermentation, optimize fertilizer use, and predict crop responses in real time. Quantum computing could be the next technological leap as agriculture enters the age of AI and data-driven tools if we are ready for it. Quantum mechanics might move from the lab bench to the barnyard, revolutionizing how we feed, breed, and manage our food systems.

## Introduction

The field of computing is on the *brink* of a paradigm shift, but perhaps not too fast. As classical computers approach the limits of Moore’s Law, quantum computing (**QC**) emerges as a revolutionary technology with the potential to solve problems intractable for even the most powerful supercomputers. Moore’s Law refers to the observation made by Gordon Moore, co-founder of Intel, in 1965 that the number of transistors on an integrated circuit doubles approximately every (one or) two years, leading to exponential growth in computing power at relatively consistent cost (Moore, 1965). This observation became a guiding principle for the semiconductor industry, driving decades of technological advancement and miniaturization. The prediction has held for many decades, though, since the 2000s, the industry has encountered physical limitations as transistors approach atomic scales and the chips get too hot (Waldrop, 2016). The physical restriction makes it increasingly difficult and costly to continue the historical pace of miniaturization, prompting semiconductor manufacturers to search for alternative computing paradigms like QC.

In physical sciences, two major scientific revolutions transformed physics in the 20th century: the theory of relativity and quantum mechanics (Aspect, 2024). Quantum physics has been recognized as having an impact comparable to the Industrial Revolution, which was powered by the laws of thermodynamics and the steam engine. As discussed later in more details, quantum physics introduces principles such as superposition (where systems can exist in multiple states simultaneously), entanglement (instantaneous correlation between particles regardless of distance), quantum interference (where probabilities combine in non-classical ways), and quantum tunneling (where particles can pass through energy barriers that would be insurmountable in classical physics). Nobel laureate Alain Aspect has noted that quantum mechanics represents one of the most profound shifts in scientific understanding in human history. Physicists typically recognize two distinct quantum revolutions: the first occurred in the early 20th century with pioneering work by Max Planck, Albert Einstein, Niels Bohr, Werner Heisenberg, and Louis de Broglie, among many others, who established the fundamental principles of quantum mechanics (Aspect, 2023, 2024). The second quantum revolution began in the 1960s–1980s, building on the theoretical work of Einstein and Erwin Schrödinger, who had identified quantum entanglement—what Einstein famously (and possibly) referred to as “spooky action at a distance”—as a profound phenomenon requiring deeper investigation (Aspect, 2023, 2024). This second revolution has led to technologies like QC, quantum cryptography, and quantum sensing that are perceived to have transformative potential across multiple fields today.

Therefore, QC harnesses the principles of quantum mechanics to process information in ways that classical computers cannot. To illustrate the potential power of QC, let’s consider a specific task: the simulation of complex molecular structures, crucial in nutritional science. For instance, simulating the behavior of a molecule like glucose, with 24 atoms (6 C, 12 H, and 6 O) and approximately 96 electrons (6 in each C, 1 in each H, and 8 in each O), would require a classical computer to track 2^96^ possible electron configurations (Aspuru-Guzik et al., 2005; Cao et al., 2019)—an astronomically large number, approximately 7.9 × 10^28^, which is about 10 billion times larger than the estimated number of grains of sand on Earth (7.5 × 10^18^) (Blatner, 2012). This task quickly becomes infeasible for classical computers as molecular complexity increases. In contrast, in theory, a quantum computer with *just* 96 quantum processing units could simulate this molecule more efficiently. The reason is that on classical computers, resource requirements for a complete simulation of the time-independent Schrödinger equation scale exponentially with the number of atoms in a molecule, limiting such full configuration interaction calculations to diatomic and triatomic molecules, whereas on quantum computers, resource requirements scale polynomially with system size (Aspuru-Guzik et al., 2005; Cao et al., 2019). The Schrödinger equation, fundamental to these simulations, describes how the quantum state of a physical system evolves over time. In its time-independent form, HΨ=EΨ, where H is the Hamiltonian operator representing total energy, Ψ is the wave function, and E represents energy levels (Griffiths and Schroeter, 2018). Classical computers struggle with this equation for complex molecules because the required computational resources grow exponentially with each additional particle, while quantum computers can potentially represent these quantum states natively using superposition and entanglement (Nielsen and Chuang, 2010).

At this pivotal juncture in computational science, QC stands as both a technological frontier and a philosophical challenge to our understanding of (quantum) information processing. While the field of quantum mechanics continues to evolve, bringing new insights that reshape our fundamental understanding of nature, QC represents its most ambitious practical application. Quantum theory stands apart from other major physical frameworks like Newtonian mechanics, Maxwell’s electrodynamics, or Einstein’s relativity in that it was not developed or definitively formulated by a single scientist and continues to bear the marks of its challenging and revolutionary origins (Griffiths and Schroeter, 2018). It seems fair to say that QC is “a technology emerging from a theory without consensus.” The science of quantum mechanics operates both mathematically and experimentally, but the fundamental meaning and interpretation of quantum mechanics remain contested among physicists and philosophers of science; it is not an easy topic to discuss on a daily basis. Nevertheless, the foundations of quantum mechanics themselves remain hotly debated in the scientific community, and no consensus on essential questions has been reached (Schlosshauer et al., 2013). This controversy is not about quantum mechanics’ mathematical formalism or experimental predictions, which are remarkably successful, but rather about what the theory implies about the nature of reality.

This review aims to bridge the theoretical with the practical by briefly explaining QC principles, contrasting them with classical computing (**CC**) paradigms, and exploring their transformative potential specifically for agricultural sciences. From simulating complex biological systems for crop improvement to optimizing resource allocation across vast agricultural networks, QC promises capabilities beyond classical limitations. Yet, as we navigate between optimistic projections and skeptical assessments, we must remain grounded in the considerable technical challenges that lie ahead. The journey toward practical “quantum advantage” will require interdisciplinary collaboration, realistic expectations, and patience as we determine whether QC will ultimately deliver on its revolutionary promise or remain constrained by fundamental physical limitations.

### Quantum computing

A quantum computer is a sophisticated device that leverages the principles of quantum mechanics to process information. Unlike classical computers that use bits (binary digits: 0 s and 1 s) to represent information, quantum computers use quantum bits or qubits. As briefly mentioned above, the power of qubits lies in four key quantum mechanical properties: superposition, entanglement, quantum interference, and quantum tunneling. Each plays a distinct role in enabling quantum computers to achieve computational capabilities that can surpass those of classical systems.

#### Superposition

**Figure 1.**
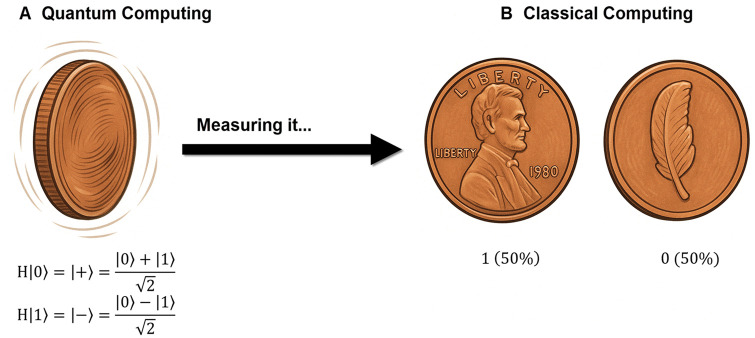
Visual analogy of quantum superposition and measurement. (A) In quantum computing, a qubit can exist in a superposition state, graphically represented by a spinning coin and mathematically described as |+⟩=(|0⟩+|1⟩)/√2 or |-⟩=(|0⟩-|1⟩)/√2, resulting from the Hadamard operation on |0⟩ and |1⟩, respectively. Upon measurement, the superposition collapses to a definite classical state (1 = heads or 0 = tails), each with 50% probability, similar to outcomes in classical computing (B).

Superposition allows a qubit to exist in multiple states simultaneously rather than discrete binary values (0 or 1). As depicted in Figure 1, a qubit in superposition is like a spinning coin that is, in a sense, both heads and tails at once until you observe it (measure it), at which point it “collapses” to just one state. It enables quantum computers to process multiple computational paths concurrently. When you have multiple qubits in superposition, they can represent all possible combinations of 0 s and 1 s simultaneously, allowing for massive parallelism in computation. This parallel processing capability provides theoretical computational advantages for specific problem classes (Preskill, 2018).

#### Entanglement

Einstein et al. (1935) described a phenomenon (later termed quantum entanglement by Schrödinger) where the quantum states of multiple particles become correlated such that the quantum state of each particle cannot be described independently. As mentioned above, Einstein later referred to this phenomenon as “spooky action at a distance” (Born and Einstein, 1971; Aspect, 2024) (translated from *spukhafte Fernwirkung*) (Hossenfelder, 2022) in his correspondence with Max Born on March 3^rd^, 1947 (Born and Einstein, 1971, p. 158). Aspect (2024) indicated that in Einstein’s opinion, “if two objects, which have interacted in the past but are now separated, present a perfect correlation, they must carry within them a set of properties determined in concert before their separation, and which then have survived in each of the objects.” This concept can be further illustrated with an analogy of homozygous twins who share identical chromosomes but live in separate countries. If these twins possess a genetic condition programmed to manifest at a specific age, both will develop symptoms simultaneously despite their geographic separation—not because of mysterious communication between them, but because they carry the exact predetermined genetic instructions (Aspect, 2024). Einstein’s discomfort with quantum entanglement led to a decades-long scientific quest to determine whether his intuition about hidden variables was correct. Bell’s (1964) theorem provided a mathematical framework to test Einstein’s local hidden variables theory against quantum mechanics. Bell derived inequalities that would be satisfied by any theory based on local hidden variables but violated by quantum mechanics in certain scenarios. Subsequent experiments, most notably those conducted by Alain Aspect and colleagues in the early 1980s, confirmed quantum mechanics’ predictions by demonstrating violations of Bell’s inequalities (Bell, 1964; Aspect et al., 1982; Aspect, 2024). These experimental results strongly suggest that quantum entanglement cannot be explained by pre-existing properties carried by particles, but represents a fundamentally different kind of correlation that defies classical intuition. Despite these philosophical challenges, entanglement has proven to be an essential resource for quantum information processing.

**Figure 2.**
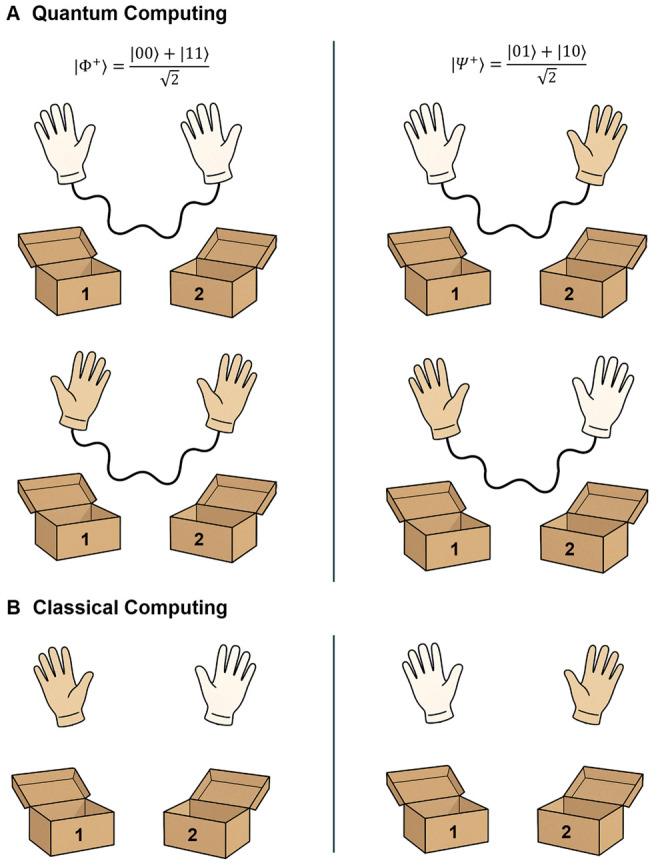
Graphical representation of quantum entanglement versus classical correlation using the glove-in-box analogy. (A) In quantum computing, two boxes contain entangled qubits represented by the quantum link (curvilinear line). On the left, the Bell state $|\Phi^{+}\rangle =(|00\rangle +|11\rangle )/\surd 2$ results in both boxes revealing the same glove upon measurement (both left or both right). On the right, the Bell state $|\Psi^{+}\rangle =(|01\rangle +|10\rangle )/\surd 2$ results in the boxes containing opposite gloves. In both cases, the outcomes are not predetermined but are correlated in a non-classical, probabilistic way. (B) In classical computing, the gloves have definite identities from the start. If one box contains a left-handed glove, the other contains a right-handed glove, but this information is only revealed when the boxes are opened. Unlike quantum entanglement, the correlation here is due to a prior assignment.

Entanglement serves as a crucial resource for quantum information processing, enabling computational capabilities beyond classical limits (Horodecki et al., 2009). It enables qubits to be correlated in ways that have no classical analog, allowing quantum computers to perform certain calculations exponentially faster than classical computers (Nielsen and Chuang, 2010). It is a quantum phenomenon where two (or more) qubits become linked in such a way that the state of one instantly affects the state of the other, no matter how far apart they are, i.e., they no longer have independent states; instead, they share a joint quantum state. In fact, Jian-Wei Pan and colleagues experimentally demonstrated that quantum entanglement persists over vast distances when, in 2017, they used the Micius satellite to distribute entangled photon pairs between ground stations separated by 1,200 kilometers, confirming that the quantum correlation remains intact regardless of spatial separation (Yin et al., 2017). As illustrated in Figure 2, the glove-in-box analogy helps introduce the concept of quantum entanglement by comparing it to a more familiar classical scenario. In the classical case, a pair of gloves—one left-handed and one right-handed—are placed in two separate boxes, which are then sealed and sent to different locations. Although the identity of the gloves is unknown until a box is opened, the glove types were fixed from the beginning. In contrast, in the quantum case, the “gloves”—representing entangled qubits—do not have definite identities prior to measurement. When one box is opened, and a glove is revealed, the glove in the other box instantaneously assumes the corresponding correlated identity, even if the boxes are far apart. Depending on the specific Bell state, a maximally entangled two-qubit state, the gloves will be either the same or opposite. For example, in the $\left|\Phi^{+}\right\rangle$ state (Figure 2A left panel), both gloves will match (e.g., both left-handed), while in the $|\Psi^{+}\rangle \;$state (Figure 2A right panel), the gloves will differ (i.e., one left-handed, one right-handed) (Mermin, 1981; Nielsen and Chuang, 2010). While Bell states represent the extreme case of maximal entanglement, in practice, entanglement can also occur to varying degrees. Although this analogy captures the idea of strong correlations and nonlocal outcomes, it does not fully reflect the unique features of quantum entanglement—such as the ability to choose different measurement settings and observe correlations that violate classical expectations (Mermin, 1981; Nielsen and Chuang, 2010). This illustrates quantum entanglement’s non-classical, probabilistic nature, where measurement outcomes are not predetermined but are perfectly correlated according to the shared entangled state. Therefore, the glove-in-box analogy is a helpful conceptual tool, but should not be interpreted as a complete description of entanglement phenomena. Another way to understand entanglement is the “quantum book” (Preskill, 2018). Unlike a classical 100-page book, where reading each page reveals 1% of the content, reading individual pages of the entangled quantum book reveals only “random gibberish.” This occurs because the information is not stored in individual pages but rather in their correlations. The quantum information can only be accessed simultaneously through collective observations of multiple pages. This characteristic distinguishes quantum information processing from CC, highlighting how entanglement allows information to be encoded in relationships between components rather than in the components themselves.

#### Quantum interference

**Figure 3.**
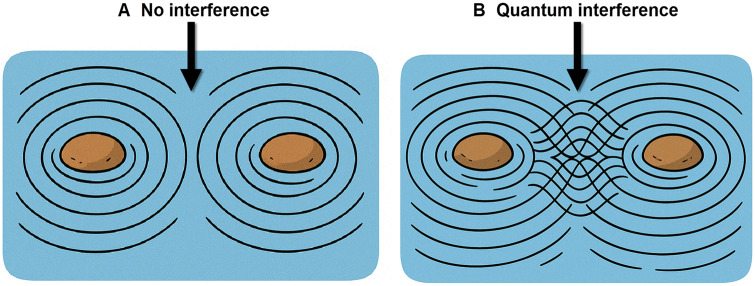
Water wave analogy for quantum interference. Ripples from two stones show (A) no interference or (B) constructive and destructive interferences, similar to how quantum algorithms amplify correct outcomes and cancel incorrect ones through the interference of probability amplitudes. While visually intuitive, this analogy has important limitations as quantum interference occurs in Hilbert space, not physical space, and involves complex amplitudes rather than physical waves (Rozenman et al., 2019).

It is the third foundational pillar of QC, alongside superposition and entanglement, and serves as the critical mechanism enabling quantum computational advantage. In the circuit model, quantum algorithms manipulate qubits using quantum gates to generate interference patterns that amplify correct outcomes and suppress incorrect ones (Nielsen and Chuang, 2010); in other paradigms, such as quantum annealing (Das and Chakrabarti, 2008), photonic continuous-variable computing (Braunstein and van Loock, 2005), and some neutral atom systems (Saffman et al., 2010), analogous interference effects are engineered through different physical processes rather than discrete gates. This selective enhancement lies at the heart of the computational speedups observed in quantum algorithms (Nielsen and Chuang, 2010; Montanaro, 2016). Quantum interference is often illustrated using the water wave analogy to build an intuitive understanding, as shown in Figure 3. When two stones are dropped into a pond, the resulting ripples overlap, producing constructive interference (when crests align) and destructive interference (when crests and troughs cancel). Analogously, quantum interference arises from the interaction of probability amplitudes, i.e., complex-valued quantities whose squared magnitudes determine the likelihood of measurement outcomes. Regardless of the implementation, quantum operations orchestrate these amplitudes to bias the probability distribution toward correct answers. However, this analogy, while helpful, is conceptually limited. Quantum and classical wave packets can share mathematical descriptions [e.g., via the Schrödinger equation; Griffiths and Schroeter (2018)], but the physical interpretations differ fundamentally (Rozenman et al., 2019). Water waves are physical displacements in a medium, whereas quantum wave functions represent probabilities and exist in an abstract Hilbert space (Rozenman et al., 2019). Hilbert space is the mathematical framework where quantum states “live”—a complete vector space with an inner product that allows for measuring distances and angles between quantum states. This abstract space is essential for QC because it provides the mathematical structure needed to describe superposition, entanglement, and the evolution of quantum systems, with its dimensionality growing exponentially with the number of qubits (2^n^ dimensions for n qubits) (Supplementary Appendix 1). The analogy breaks down especially when interpreting interference as a purely spatial effect; quantum interference operates in the space of possibilities, not classical geometry. This distinction is crucial: quantum interference is not merely a wave phenomenon but a non-classical computational resource that allows algorithms like Grover’s search and quantum Fourier transform to outperform their classical counterparts (Rozenman et al., 2019).

While superposition, entanglement, and quantum interference form the core of most discussions on QC, certain qubit architectures rely on an additional quantum phenomenon, quantum tunneling, as a fundamental operational resource.

#### Quantum tunneling

**Figure 4.**
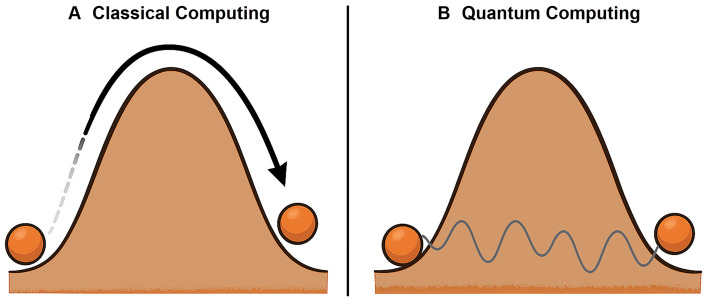
Illustration of quantum tunneling using the hill and the ball analogy. In classical computing, a ball on one side of a hill cannot cross to the other side without enough energy to roll over the top. In contrast, in quantum computing, the same ball “mysteriously” appears on the other side without ever going over the top because the particle’s wavefunction can extend through the barrier (i.e., hill), giving a finite probability that the particle will appear on the other side without traversing over the top.

Quantum tunneling occurs when a particle passes through an energy barrier that it would be unable to overcome according to classical physics. This effect arises from the wave-like nature of quantum particles and the probabilistic nature of their wavefunctions (Griffiths and Schroeter, 2018). The concept can be visualized by imagining a ball resting at the base of a slope that is too steep for it to climb; while classical mechanics predicts the ball will remain trapped, quantum mechanics allows it to be found on the other side without ever cresting the slope, due to its wavefunction extending into and beyond the barrier ­(Figure 4). In superconducting qubits, paired electrons known as Cooper pairs can tunnel through an ultra-thin insulating barrier in a Josephson junction. This tunneling is central to the operation of the qubit: it creates discrete, anharmonic energy levels that can be manipulated to represent and process quantum information (Clarke and Wilhelm, 2008; Krantz et al., 2019). By enabling precise and coherent control of these states, tunneling complements superposition, entanglement, and quantum interference as a fundamental quantum resource in specific qubit architectures.

Together, superposition, entanglement, quantum interference, and quantum tunneling form the foundational quantum phenomena that enable computation in fundamentally different ways than classical computers. While CC algorithms are sequences of logical operations performed on bits, quantum algorithms manipulate qubits through quantum gates, exploiting these quantum phenomena to perform specific calculations more efficiently than classical algorithms (Arute et al., 2019). Current quantum computers use various physical systems to implement qubits, including superconducting circuits, trapped ions, and topological systems. Each approach has advantages and challenges, and research is ongoing to determine the most effective and scalable qubit technologies for each application (Bennett and DiVincenzo, 2000). The potential of QC is perhaps best illustrated by Shor’s quantum algorithm for integer factorization, which can theoretically break many current cryptographic systems. While factoring large numbers is computationally intensive for classical computers, Shor’s algorithm could factor them exponentially faster. This poses significant implications for cybersecurity (Shor, 1997) as it could compromise widely used encryption methods like RSA (Rivest–Shamir–Adleman), the most common public-key cryptographic algorithm used today. The RSA’s key exchange security relies on the practical difficulty of factoring a large semiprime number—i.e., a product of two large prime numbers. Classically, this requires checking potential prime factors up to the square root of the target number, a task that becomes computationally infeasible as the number of digits increases (Aspect, 2024).

### Evolution of quantum computing

**Figure 5.**
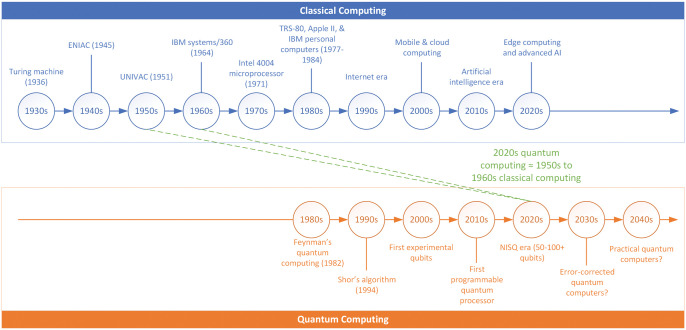
Comparative evolutionary timeline of classical and quantum computing (1930s to 2040s).

Contextualizing QC progression through comparison with historical CC development provides a valuable perspective. Figure 5 illustrates the parallel but offset developmental trajectories of classical and QC technologies. While CC has evolved from theoretical concepts to ubiquitous systems over nearly a century, QC remains in its early stages with capabilities comparable to those of CC in the 1950s-–1960s. Major technological milestones are indicated for each paradigm, with projected developments for QC extending into the 2030s and 2040s. The green dashed lines highlight the developmental equivalence between 2020s QC and 1950s–1960s CC.

#### Classical computing developmental timeline

The evolution of CC unfolded as a remarkable technological odyssey spanning nearly half a century (Ceruzzi, 2003). Beginning with Alan Turing’s theoretical foundations in 1936 that conceptualized the universal computing machine (Turing, 1937), this journey gained physical form with the Electronic Numerical Integrator and Computer’s (**ENIAC**) completion in 1945, the first programmable, electronic, general-purpose digital computer. However, ENIAC was not a stored-program machine; it required manual reconfiguration using cables and switches to change programs. The first successful implementations of the stored-program concept appeared shortly after, with the Manchester Baby prototype in 1948 and the Electronic Delay Storage Automatic Calculator (**EDSAC**) in 1949, which is widely recognized as the first computer to run a full program stored in memory. The commercial computing era truly began when Universal Automatic Computer I (**UNIVAC**) was delivered to the U.S. Census Bureau in 1951, establishing the feasibility of business-oriented data processing. The revolutionary IBM System/360, announced in 1964, introduced the first standardized computer family, allowing businesses to upgrade their computing capabilities without rewriting applications and cementing IBM’s dominance in the industry. The microprocessor revolution followed with Intel’s 4004 in 1971, dramatically shrinking computing power onto a single chip and paving the way for personal computing’s emergence through a watershed moment in 1977 when three pioneering “trinity” systems launched: the Apple II, Commodore PET, and Tandy Radio Shack (**TRS**-80), collectively bringing affordable computing to homes and small businesses for the first time. The TRS-80 model I was particularly important as it was one of the first mass-produced, fully assembled personal computers available through widespread retail distribution (i.e., RadioShack), making computing accessible to many Americans who didn’t have access to specialized computer stores (Welsh and Welsh, 2007). The introduction of the IBM PC in 1981 further accelerated adoption and standardization. Subsequent decades witnessed exponential growth in computing power, alongside the transformative impacts of networking, mobile devices, and sophisticated software systems, culminating in CC’s journey from esoteric research tools to ubiquitous global infrastructure (Ceruzzi, 2003), including supercomputers.

#### Quantum computing developmental timeline

The QC paradigm represents a fundamentally different technological trajectory still in its embryonic phase compared to its classical counterpart (Figure 5). This journey began when Richard Feynman publicly articulated the concept during a 1981 conference at MIT, proposing that quantum systems could be effectively simulated only by computers leveraging quantum mechanical properties (Feynman, 1982). As Georgescu et al. (2014) later confirmed, quantum computers possess an inherent advantage in modeling other quantum systems—a task that becomes exponentially complex on classical architectures. This capability holds transformative potential for materials science and pharmaceutical research, leading to the development of quantum chemistry, an intersection among physics, chemistry, computer science, and applied mathematics (Aspuru-Guzik et al., 2018). The field gained theoretical rigor when David Deutsch formalized the universal quantum computer model in his seminal 1985 paper, establishing the mathematical foundation for quantum computation (Deutsch, 1985). Practical algorithms emerged in the mid-1990s through groundbreaking work by Peter Shor (Shor, 1994) and Lov Grover (Grover, 1996), demonstrating QC’s potential superiority for specific problems. Experimental implementation has progressed gradually from rudimentary qubits in the early 2000s to today’s noisy intermediate-scale quantum (**NISQ**) systems with 50 to 100+ qubits (Preskill, 2018). NISQ refers to the current generation of quantum computers that operate with a moderate number of qubits (typically below 1,000) but suffer from significant noise and quantum decoherence (i.e., the loss of quantum information due to unavoidable interactions with the environment), limiting their ability to maintain quantum states and perform error-free calculations (Preskill, 2018). These systems lack the comprehensive error correction needed for fault-tolerant computation, yet they are sufficiently powerful to explore quantum algorithms and potentially demonstrate quantum advantage in specific applications. However, practical quantum advantage remains elusive for most applications.

While decoherence remains a fundamental limitation for maintaining quantum states over the durations required for computation, the current biggest obstacle to practical quantum advantage is the limited number of physical qubits available in existing systems, a challenge that decoherence and other noise sources exacerbate by constraining how many logical qubits can be derived from physical ones. For many commercially relevant problems, the scale of the computation exceeds the capacity of today’s devices, even before accounting for the overhead required for error correction. Quantum computation requires qubits to interact with external control systems to perform operations such as entangling gates. These necessary interactions inevitably expose the system to environmental noise, contributing to decoherence. This creates a fundamental tension: qubits must remain sufficiently isolated to preserve coherence, yet accessible enough to perform logic operations (Black et al., 2002). Recent experiments show typical coherence times of 50 microseconds to a few milliseconds for superconducting qubits (Burnett et al., 2019), far shorter than required for complex algorithms. Error correction requires significant overhead, with estimates suggesting that thousands of physical qubits are needed for each logical qubit capable of fault-tolerant operation (Fowler et al., 2012). Current error rates for two-qubit gates remain at approximately 0.5% to 1% for leading platforms (Noiri et al., 2022), whereas fault tolerance generally requires error rates below 0.1% (Wang et al., 2011). These constraints make hybrid quantum–classical approaches essential in the near term, with quantum processors focused on the subproblems where they offer the greatest advantage, while classical computers handle the remaining computation.

As shown in Figure 5, QC development currently approximates the evolutionary stage that CC occupied during the 1950s–1960s period, which is characterized by early implementations with limited functionality but substantial future potential. The theoretical and applied QC fields are rapidly gaining traction, with significant advancements likely to occur sooner rather than later. Most industry experts and researchers project that error-corrected quantum systems could be achieved within the next decade, with a possible fault-tolerant, practical QC implementation after 2025. In stark contrast to this optimistic outlook, Dyakonov (2020) presents a fundamentally skeptical assessment of QC’s future. He argues that QC faces insurmountable physical challenges rather than merely engineering obstacles to be overcome with time. He contends that controlling the vast number of continuous quantum parameters required (which grows exponentially with the number of qubits) is physically impossible in practice. Furthermore, he asserts that quantum error correction schemes cannot work as theorized because they rely on mathematical abstractions that ignore fundamental physical realities. In his assessment, after 25 years of research with no meaningful computational results, QC represents “more of a sociological phenomenon than a viable technological path” (Dyakonov, 2020).

The “quantum supremacy” concept represents an important milestone in developing QC technology. In its conventional definition, quantum supremacy refers to the point at which a quantum computer can perform a well-defined computational task that is practically impossible for classical computers to complete within a reasonable timeframe (Yung, 2019). It is important to distinguish this from “quantum advantage”: supremacy, in the traditional sense, can apply to any task performed faster by a quantum computer, even if the task has no direct practical value (as in Google’s 2019 random circuit sampling experiment described next), whereas quantum advantage refers to solving practical, real-world problems more efficiently than classical systems. Some researchers have proposed a broader interpretation of supremacy that is not limited to execution speed, emphasizing that quantum systems may also offer unique representational capabilities, for example, in quantum machine learning (**QML**), where projecting classical data into high-dimensional Hilbert spaces can capture correlations inaccessible to classical computation, even if the quantum approach is slower. For agricultural applications (and likely many other applied sciences), our ultimate focus is on achieving quantum advantage, i.e., practical benefits for real-world problems, rather than merely demonstrating supremacy.

### Current state of quantum computing

Despite the considerable media hype surrounding QC, including Google’s landmark 2019 quantum supremacy claim that their 53-qubit Sycamore processor performed a specialized calculation in 200 seconds that they estimated would take a classical supercomputer 10,000 years, causing them to declare “an experimental realization of quantum supremacy for this specific computational task” (Arute et al., 2019). Google’s claim was immediately challenged by IBM researchers, who demonstrated that by leveraging secondary storage on the Summit supercomputer, such circuits could be simulated “with high fidelity to arbitrary depth in a matter of days” (Pednault et al., 2019), effectively reducing Google’s estimated 10,000 years to approximately 2.5 days.

As of 2025, QC has progressed from a theoretical concept to an emerging technology with significant potential. Given Google’s 2019 premature announcement of quantum supremacy, it is important to note that the QC market is currently highly volatile, with rapidly changing claims, counterclaims, and projections from various industry players and researchers. It is crucial to approach industry announcements with appropriate scientific skepticism (Roberson and White, 2019). While large-scale, fault-tolerant quantum computers (**FTQC**), i.e., systems with comprehensive error correction capabilities that can reliably perform quantum computations despite noise and decoherence, remain elusive, several milestones have been reached. Companies like IBM, Google, and IonQ have developed quantum processors with increasing numbers of qubits. For instance, IBM’s Osprey processor, announced in 2022, featured 433 qubits (Collins and Nay, 2022), though the quantum volume, a hardware-agnostic metric that measures the performance of quantum computers by considering both qubit count and error rates (Moll et al., 2018), remains significantly lower than the raw qubit count would suggest. Unlike raw qubit count, which tallies the number of qubits, quantum volume captures the *effective* computational power by incorporating critical factors such as gate fidelity, connectivity between qubits, and circuit depth capabilities. For example, a 100-qubit processor with poor connectivity and high error rates might have a lower quantum volume than a 20-qubit processor with all-to-all connectivity and low errors, making the quantum volume a more realistic measure of what computations can actually be performed successfully.

Then, in late 2023, IBM announced its Condor processor, featuring 1,121 qubits and representing the world’s largest quantum chip at the time (Pasternack, 2024). However, Condor also demonstrated the practical limits of simply scaling up qubit count, as the massive chip required unprecedented cooling infrastructure and generated significant engineering challenges related to noise, connectivity, and error rates. Simultaneously, IBM developed their Heron processor (133 qubits), which prioritizes error mitigation and gate fidelity over raw qubit numbers, alongside a modular system architecture designed to link multiple high-quality processors together (Pasternack, 2024). In 2024, IBM released the Heron R2, expanding to 156 qubits with improved coherence times and the ability to execute up to 5,000 two-qubit gates, enabling what IBM terms “utility-scale” quantum computation (Ivezic, 2024). In 2025, IBM unveiled the Nighthawk processor (120 qubits), featuring a novel square lattice connectivity map designed to improve nearest-neighbor coupling and reduce circuit depth. Although Nighthawk’s qubit count is lower than Condor or Osprey, its architecture supports up to ∼15,000 gates and can scale by chaining up to nine modules (∼1,080 qubits) while maintaining high fidelity (Anonymous, 2025b). Looking further ahead, IBM announced its goal to deliver the Starling quantum supercomputer by 2029, projected to contain ∼2,000 physical qubits and 200 logical qubits with full error correction, capable of executing approximately 100 million quantum gates—marking a potential transition to scalable, fault-tolerant quantum computation (Tatananni, 2025). IBM’s dual development of Condor’s brute-force scaling approach and Heron’s quality-focused design exemplifies the broader industry recognition that quantum advantage will likely emerge from improved qubit coherence and error correction rather than simply maximizing qubit count. The addition of Nighthawk and Starling to IBM’s roadmap reinforces this hybrid strategy, combining near-term gains from improved connectivity and modular scaling with long-term fault-tolerant system goals.

In February 2025, Amazon Web Services (**AWS**) unveiled its inaugural quantum processor, designated “Ocelot” (Anonymous, 2025a). This superconducting qubit-based processor incorporates novel error correction methodologies designed to “reduce the costs of implementing quantum error correction by up to 90%, compared to current approaches.” Several companies now offer cloud access to quantum computers, allowing researchers and developers to experiment with quantum algorithms (Preskill, 2018). Researchers have made critical advances in quantum error correction, a crucial step towards FTQC. For example, a 2023 study demonstrated significant progress in developing practical quantum error correction methods (Fowler et al., 2012; Campbell, 2024), outperforming its constituent physical qubits (Kim et al., 2023) and representing a significant threshold in the field. However, considerable challenges remain, including improving qubit quality and quantity, developing practical quantum algorithms, and creating a robust quantum software ecosystem (Preskill, 2018).

Perspectives on QC timelines vary substantially across the industry. Their expert projections in academic and industry analyses indicate that truly FTQC capable of solving practical problems will likely emerge around 2030 (Bobier et al., 2024), though some companies are accelerating their timelines with targeted roadmaps (Baker, 2024). Some suggest that truly FTQC capable of delivering reliable, practical business value will not emerge soon; expert projections indicate that “everyday quantum computers are still decades away” as there remains “a vast gap to be bridged before quantum computers can do more meaningful things” (Anonymous, 2019). According to comprehensive market analyses by Boston Consulting Group (**BCG**), the QC industry is expected to develop in three distinct phases: NISQ (i.e., computers with less than 1,000 qubits) until 2030, broad quantum advantage from 2030 to 2040, and full-scale fault tolerance after 2040 (Bobier et al., 2021). This phased development reflects the significant technical challenges in quantum error correction that experts believe will remain QC’s biggest hurdle for much of this decade (Preskill, 2023). The timeline projections of QC exhibit substantial variation among domain experts (Novet, 2025): Oskar Painter, AWS Director of Quantum Hardware, projected that commercial quantum workloads will not be operational for “10 years or more.” Similarly, Jensen Huang, Nvidia CEO, estimated that practical QC applications remain “15 to 30 years” from realization, while Mark Zuckerberg, from Meta, suggested a minimum developmental timeline of “at least a decade” before commercial viability. Contrastingly, Julian Kelly, Google Quantum Artificial Intelligence (**AI**) Director of Hardware, presented a more optimistic assessment, suggesting QC technology is “about five years out from a real breakout application that you can only solve on a quantum computer” (Cherney, 2025). McKinsey’s quantitative analysis estimated that approximately 5,000 operational quantum computers would be in place by 2030, with hardware and software capabilities sufficient for complex computational problems not anticipated until 2035 or beyond (Stackpole, 2024).

While we are currently in the NISQ era, as first defined by Preskill (2018), recent advances suggest we may see “early fault-tolerant quantum computers” with a few hundred logical qubits in the latter half of this decade. These early FTQC systems will enable practical applications but are not yet powerful enough to run intensive quantum algorithms like Shor’s factorization (Anonymous, 2024a). Multiple industry leaders, including Quantinuum, have published roadmaps targeting FTQC by 2030 (Anonymous, 2024b), demonstrating progress through achievements like creating 12 logical qubits on existing hardware. These developments support BCG’s projection that QC will make $450–850 billion of economic value by 2040, with the most transformative commercial applications requiring the broad quantum advantage expected in the 2030–2040 timeframe (Bobier et al., 2024).

The field has recently seen significant progress in intermediate-scale applications that do not require complete fault tolerance. Variational quantum algorithms have shown promise in chemistry simulations (McArdle et al., 2020), though they still face limitations in accuracy compared to classical methods. Additionally, QML approaches are being developed that may offer advantages for specific data structures (Biamonte et al., 2017), but the general quantum advantage for practical problems remains unproven experimentally. According to the National Academies of Sciences, Engineering, and Medicine (NASEM, 2019), the scientific consensus suggests that while QC holds tremendous potential, the path to reliable, practical business value requires overcoming substantial technical barriers and may take 5-10 years of further fundamental research.

### Classical versus quantum computing

#### Fundamental differences

The field of computing stands at a fascinating crossroads, with QC emerging as a revolutionary paradigm that challenges our traditional understanding of information processing. Table 1 summarizes the key differences between CC and QC. The fundamental distinction between them begins at the most elementary level of information representation (Nielsen and Chuang, 2010). As indicated before, while classical systems rely on bits that exist in definitively binary states (0 or 1), quantum computers leverage quantum mechanical phenomena to utilize qubits, which can exist in superpositions of states (Preskill, 2018). This fundamental difference cascades into profound operational distinctions. Classical computers process information sequentially through Boolean logic operations on discrete binary states. In contrast, quantum systems can perform parallel operations on exponentially large state spaces (Nielsen and Chuang, 2010). Classical systems benefit from relatively straightforward error correction mechanisms, whereas quantum systems remain exquisitely sensitive to environmental perturbations—a phenomenon known as decoherence (Zurek, 2003). This sensitivity necessitates sophisticated quantum error correction codes, which require significant overhead to implement effectively (Terhal, 2015). Recent advances in error correction have been substantial, with Google demonstrating the first “below-threshold” error correction in 2024 using their 105-qubit Willow processor, achieving exponential error suppression with increasing code size (Acharya et al., 2025). Perhaps most significantly, the algorithmic approaches diverge fundamentally. While classical algorithms rely on deterministic or probabilistic operations rooted in Boolean logic, quantum algorithms exploit unique quantum phenomena such as interference and entanglement to achieve computational advantages for specific problems (Montanaro, 2016).

**Table 1.** Key differences between classical and quantum computing

Aspect	Classical Computing (CC)	Quantum Computing (QC)
**Information units**	Bits (0 or 1)	Qubits (superposition of 0 and 1)
**State representation**	Binary states	Superposition of states
**Processing**	Sequential operations on bits	Parallel operations on quantum states
**Error susceptibility**	More stable, easier to correct errors	Highly sensitive to environmental interference, requires complex error correction
**Algorithmic approach**	Based on boolean logic and arithmetic	Exploits quantum phenomena like interference and entanglement

#### Comparative advantages and limitations

Recent advances in Shor’s algorithm implementation have been notable, with researchers successfully factoring 21 on IBM quantum processors using only five qubits (Skosana and Tame, 2021). The largest number factored using a complete simulation of Shor’s algorithm on classical hardware without prior knowledge of the solution is 549,755,813,701 (Willsch et al., 2023), though practical implementation on quantum hardware remains challenging for larger numbers. One particularly promising application domain involves the simulation of quantum systems. Other advantages include the quantum approximate optimization algorithm (**QAOA**) (Farhi et al., 2014; Blekos et al., 2024) and quantum annealing techniques (Hauke et al., 2020) that demonstrate promising theoretical advantages for complex optimization problems that pervade scientific and industrial domains. The intersection of QC with machine learning (**ML**) represents another frontier with significant potential, as QML algorithms may offer substantial speedups for specific learning tasks and data structures (Biamonte et al., 2017; Schuld and Petruccione, 2021). However, QC is not universally superior—many computational tasks show no quantum advantage and remain better suited to classical architectures (Aaronson, 2015). As mentioned above, current quantum error correction methods require significant overhead, with estimates suggesting thousands of physical qubits needed for each logical qubit capable of fault-tolerant operation (Campbell, 2024). This massive overhead is necessary because quantum states are extremely fragile, as any interaction with the environment can cause errors. Error correction codes work by encoding the information of one “logical” qubit across many “physical” qubits in a way that allows detection and correction of errors without directly measuring (and thus destroying) the quantum information. The more physical qubits used, the more robust the logical qubit becomes against noise and decoherence, but this comes at the cost of needing perhaps 1,000–10,000 physical qubits to create just one reliable logical qubit. Developing effective quantum algorithms presents another substantial hurdle because creating algorithms that leverage quantum effects to outperform classical approaches requires fundamentally different problem-solving paradigms (Montanaro, 2016). Additionally, current QC infrastructure remains costly and specialized, limiting accessibility and widespread adoption (NASEM, 2019).

#### Performance comparison

When considering performance, the relative advantage of QC requires nuanced analysis. For specific problems with appropriate structure, quantum computers theoretically offer exponential improvements in computational efficiency, but for many common computational tasks, classical computers remain not only adequate but superior in terms of both performance and practicality (Harrow and Montanaro, 2017). The distinctive value proposition of QC lies not in raw processing speed but in algorithmic efficiency for particular problem classes. As mentioned above, the so-called quantum supremacy (Arute et al., 2019; Zhong et al., 2020) is purely of theoretical significance but possibly hints at the technology’s future potential. The achievements of Google’s Willow processor represent a critical step toward building noise-resistant quantum computers with practical scale and have demonstrated significant progress in quantum error correction, achieving a “beyond-classical” ability in random circuit sampling tasks that would be intractable for classical supercomputers (Acharya et al., 2025).

#### The black box example

To better understand how quantum computers perform calculations differently from classical computers, let’s consider a simple problem and compare the approaches. Imagine a black box function that takes a single bit as input and produces a single bit as output. The function is either constant (always outputs zero or always outputs one: f(0) = f(1) = 0 or f(0) = f(1) = 1) or balanced (outputs 0 for half the inputs and 1 for the other half: either f(0) = 0, f(1) = 1 or f(0) = 1, f(1) = 0). Our task is to determine which type of function we have. As shown in Supplementary Appendix 1, a classical computer must check the function twice to be certain. Step 1: calculate f(0), step 2: calculate f(1), and step 3: compare both outputs. The function is constant if f(0) = f(1). The function is balanced if f(0) ≠ f(1). Using Deutsch’s algorithm (Deutsch, 1985), a quantum computer can solve this problem with only one function evaluation. The ingenious aspect of Deutsch’s algorithm is in how it uses superposition to evaluate both inputs simultaneously (quantum parallelism), encodes the result in phase rather than directly reading output values (phase kickback), and uses interference through the final Hadamard gate to extract global information about the function without determining specific values. Phase kickback is a quantum phenomenon where information about a function gets encoded in the phase (the quantum mechanical ‘angle’) of a qubit rather than its amplitude. This is crucial because while we cannot directly measure phase, we can use quantum gates to convert phase differences into measurable probability differences, allowing us to extract global properties of functions with fewer evaluations than classically possible. This demonstrates a fundamental quantum advantage: the ability to extract global properties of a function without evaluating all possible inputs individually, something provably impossible in CC (Mermin, 2007).

### Emulation of quantum computing

Although it is possible to simulate quantum computations on classical computers to a certain extent using Python libraries such as Qiskit, Cirq, PennyLane, and many others (Aleksandrowicz et al., 2019; Kaiser and Granade, 2021; Young et al., 2023), it is crucial to understand the distinction between quantum simulators and quantum emulators. Simulators perform idealized, noise-free quantum circuit simulations, allowing researchers to test algorithms without hardware imperfections. Some simulators, such as IBM’s Aer framework (https://qiskit.github.io/qiskit-aer), also allow the injection of custom or hardware-derived noise models to mimic real-world conditions (Aleksandrowicz et al., 2019). Emulators, on the other hand, replicate the behavior of a specific quantum processing unit, including its native gate set, connectivity, and calibrated noise distributions, often using snapshots of the device’s state at a given time. This makes emulators valuable for assessing algorithm performance under realistic hardware constraints before running on actual quantum devices.

Classical computers can effectively simulate quantum systems with a small number of qubits (typically up to about 30–40 qubits) (Gangapuram et al., 2024). Beyond this, the simulation becomes exponentially more resource-intensive due to the vast state space that needs to be represented (Zhou et al., 2020). Simulators and emulators are invaluable for developing and testing quantum algorithms, educating students and researchers, and exploring quantum concepts. However, they cannot replicate the full power of a quantum computer for large-scale problems. The exponential advantage of quantum computers for specific tasks only becomes apparent when dealing with issues beyond the simulation capabilities of classical computers (Preskill, 2018). For example, while a classical computer can simulate Shor’s algorithm for small numbers, it cannot do so for the large numbers used in real-world cryptography—that’s where actual quantum hardware becomes necessary (and troublesome). In essence, QC emulation on classical computers serves as a crucial bridge in developing QC, but it does not negate the need for actual quantum hardware to realize the full potential of QC.

## Current Development Paradigms and Limitations

Despite significant advances in QC research, fully functional large-scale quantum computers do not yet exist. These systems, while impressive demonstrations of quantum principles, remain limited in their practical capabilities due to issues with qubit coherence, error rates, and scaling. We are currently at a critical juncture in QC development. Classical computers can simulate quantum systems up to a specific size, but, as discussed before, we are approaching the “quantum advantage” or “quantum supremacy” era previously introduced (Yung, 2019). This significant threshold marks where QC hardware must progress independently as classical emulation becomes computationally infeasible. Three primary approaches are currently being pursued to demonstrate quantum supremacy: boson sampling, sampling from instantaneous quantum polynomial circuits, and sampling from chaotic quantum circuits (Yung, 2019). Each of these methods represents a pathway to establishing the practical superiority of quantum systems for specialized computational tasks. Applying these quantum supremacy techniques to agricultural problems would represent a transformative capability for addressing previously stubborn challenges in the field.

### The quantum assembly language era

The current state of QC bears striking similarities to the early days of CC when assembly language was the primary programming method (Figure 5). Today’s quantum developers work directly with low-level qubit manipulations, quantum gates (i.e., fundamental operations of qubits), and circuit designs, essentially the “assembly language” of QC. We are still developing the basic building blocks before more abstract, user-friendly QC languages and frameworks emerge. While several quantum programming languages already exist, such as IBM’s Qiskit (Aleksandrowicz et al., 2019), Google’s Cirq, and Microsoft’s Q# (Kaiser and Granade, 2021), most gate-based systems ultimately compile down to OpenQASM, an open quantum assembly language used across multiple vendors (Cross et al., 2017). They still operate at a relatively low level of abstraction compared to modern classical programming languages, often require programmers to think about quantum circuits, gates, and physical qubit operations rather than high-level abstractions. This state of development means quantum algorithms often require deep expertise in quantum mechanics and low-level quantum operations, creating a significant barrier to entry for domain specialists in fields like agriculture who might benefit from quantum applications. Furthermore, as our theoretical and experimental understanding of quantum systems evolves, our current programming models—rooted in classical logic and sequential execution—may prove insufficient to fully capture or exploit the inherently non-classical nature of quantum information, such as entanglement and quantum interference. This suggests that future breakthroughs may involve better tools and new ways of conceptualizing algorithm design and computation.

Among the various physical approaches to implementing quantum computers, neutral atom systems show particular promise for scalability. These systems can potentially prepare qubit arrays in one, two, or three-dimensional geometries, with recent experiments demonstrating control of up to 50 atomic qubits (Saffman, 2019). The high ratio between coherent coupling (the rate at which quantum operations can be performed) and decoherence (i.e., unwanted loss of quantum information) in neutral-atom systems establishes a favorable foundation for scalability. This scalability will be essential for reaching the million-qubit threshold that meaningful agricultural applications may eventually require.

### Current application development approaches

Given these hardware limitations, QC applications are currently developed through three main approaches: classical emulation (as discussed above), hybrid classical-quantum approaches, or limited-scale quantum hardware. Researchers use classical computers to simulate quantum algorithms for small problem sizes in the classical emulation. These emulators become exponentially slower as the number of qubits increases, effectively limiting simulations to about 30–40 qubits on the most powerful supercomputers. The hybrid classical-quantum approach uses classical computers to handle parts of the problem while offloading specific computations to quantum processors (Bharti et al., 2022; Cerezo et al., 2022). This approach works within hardware constraints while leveraging quantum advantages for suitable sub-problems. In the limited-scale quantum hardware, researchers access actual quantum processors through cloud services provided by companies like IBM, Google, Amazon, and others. These systems allow testing of real quantum algorithms but with significant constraints on qubit count, coherence time, and error rates.

Agriculture presents a unique confluence of computational challenges that align remarkably well with QC’s strengths. Unlike many industrial applications that involve straightforward optimization, agricultural systems encompass quantum-mechanical processes at the molecular level (soil chemistry, photosynthesis, nitrogen fixation), exponentially complex optimization problems (resource allocation across time, space, and uncertain weather conditions), and massive multivariate datasets from genomics, phenomics, and environmental sensors. These characteristics—quantum processes, combinatorial explosion, and high-dimensional data—precisely represent the domains where quantum computers promise advantages over classical systems. Furthermore, agriculture’s pressing need for sustainability solutions demands computational breakthroughs to optimize resource use, minimize environmental impact, and feed a growing global population within planetary boundaries. To illustrate the potential alignment between agricultural computational challenges and QC capabilities, Table 2 summarizes key application areas across molecular simulations, optimization problems, genomics, data analysis, and integrated systems. While these applications show theoretical promise, it is crucial to recognize that practical implementation awaits significant advances in quantum hardware, as discussed throughout this review.

**Table 2.** Potential quantum computing applications in agricultural sciences.^1^

Agricultural challenge	Classical computing (CC) limitation	Quantum computing (QC) approach	Potential impacts
**Molecular-level simulations**			
** Nitrogen fixation and soil chemistry**	Exponential scaling with molecular size; cannot model quantum effects	Quantum simulation at molecular level using Hamiltonian approaches	More efficient fertilizers and sustainable soil management
** Rumen fermentation modeling**	Cannot capture quantum-level interactions among microbes, compounds, and host	Simulate quantum interactions in microbial communities	Optimize feed efficiency, reduce methane emissions
**Optimization problems**			
** Resource allocation (water, crops)**	NP-hard problems with exponential combinations	QAOA for combinatorial optimization	Optimal resource distribution across farms
** Supply chain management**	Perishable products create NP-hard optimization problems	Quantum algorithms for multi-objective programming	Reduced waste, improved logistics
** Feed delivery routing**	Classical heuristics are suboptimal for multiple barns	QAOA for route optimization	Reduced labor costs, timely feeding
**Genomics and breeding**			
** Genetic evaluation**	O(n³) scaling; days of processing for millions of animals	Harrow-Hassidim-Lloyd algorithm (with limitations noted)	Potentially faster processing
** Genome assembly**	O(N) search complexity for DNA fragments	Grover’s algorithm: O(√N) speedup	Faster assembly of indigenous breed genomes
**Data analysis and machine learning**			
** Satellite image classification**	Limited by sequential processing	Quantum-enhanced machine learning	Improved land-use analysis
** PLF sensor data analysis**	High-dimensional data from multiple sources	Quantum neural networks, quantum kernel methods	Real-time behavioral predictions
** Climate modeling**	Multiple variables processed sequentially	QC’s parallel processing capability	Better on-farm decisions
**Integrated systems**			
** Digital twins for livestock**	Limited fidelity with CC	Quantum-enhanced simulations	Real-time optimization of management
** Crop-livestock integration**	Complex nutrient flow optimization	Quantum optimization algorithms	Whole-farm efficiency

1 NP-hard = nondeterministic polynomial time optimization problems. O(n³) refers to computational complexity notation indicating that the time required to solve the problem increases with the cube of the input size (n). For genetic evaluation, this means that doubling the number of animals (n) would increase computation time by approximately 8-fold (2³), making large-scale evaluations computationally intensive. O(N) indicates linear time complexity, where runtime is proportional to input size N. O(√N) indicates square root scaling achieved by Grover’s quantum search algorithm. For genome assembly with N DNA fragments, this means that quantum search could reduce 1 million classical operations to just 1,000 quantum operations, resulting in a quadratic speedup. QAOA = quantum approximate optimization algorithm.

## Applications in Agricultural Sciences

Given the current stage of development of QC, the most probable applications within the next 5- to 10-year timeframe (Bharti et al., 2022) include quantum chemical simulation of molecular structures and reactions where quantum mechanical effects significantly influence system behavior, particularly relevant for pharmaceutical development and materials science (McArdle et al., 2020), optimization of specialized problems using combinatorial optimization applications in logistics, supply chain management, and financial portfolio construction where current heuristic approaches demonstrate suboptimal performance (Harrigan et al., 2021), and applications to advancing theoretical understanding of quantum algorithms, error correction methodologies, and computational complexity classifications (Aaronson, 2015). For most commercial organizations and individual consumers, QC benefits will initially manifest indirectly through improved products, materials, and services rather than through direct quantum computational interaction (NASEM, 2019).

### Fundamental sciences

Biology and chemistry are perhaps the most foundational scientific disciplines poised to benefit significantly from QC due to their reliance on complex quantum mechanical interactions that are difficult to simulate classically. Quantum computing offers unprecedented potential for accurately modeling molecular electronic structures and solving the Schrödinger equation (Nielsen and Chuang, 2010; Griffiths and Schroeter, 2018) for large, interacting systems, tasks that are infeasible with classical methods (Cao et al., 2019; Baiardi et al., 2023). In chemistry, QC enables more precise calculations of ground and excited states of molecular systems using algorithms like quantum phase estimation and variational quantum eigensolvers, which are especially relevant in areas like catalyst design and reaction mechanism elucidation (Cao et al., 2019; McArdle et al., 2020). Complex configurational searches in materials like graphene can be reformulated into quantum-amenable optimization problems using quantum annealing (Camino et al., 2023). In biology, QC is expected to revolutionize areas such as drug discovery, where quantum algorithms could vastly improve the prediction of binding affinities, protein folding, and the mapping of biochemical interactions at atomistic resolution (Baiardi et al., 2023).

### Agricultural sciences

A critical question for agricultural research is whether QC offers solutions to fundamental (existing) problems that CC systems, including supercomputers and AI, cannot resolve. While CC continues to advance agricultural sciences through ML models, digital farm twins, and complex optimization algorithms (Tedeschi, 2019), particular agricultural challenges may benefit from quantum approaches. Recent comprehensive reviews have identified several promising QC applications across agricultural and life sciences domains, including bioinformatics, remote sensing, climate modeling, and smart farming (de Souza et al., 2024; Pook et al., 2025).

Quantum computers could potentially provide unique advantages in complex molecular simulations, such as modeling nitrogen fixation and soil chemistry processes at the quantum level through Hamiltonian simulation approaches for resource recovery from agricultural waste streams, enabling the development of more efficient fertilizers and sustainable soil management strategies towards sustainability. These molecular simulations involve quantum mechanical interactions that classical computers struggle to model accurately. Quantum algorithms such as QAOA might better handle the exponentially complex optimization problems in large-scale agricultural planning involving numerous variables like water usage, crop rotation, and resource allocation, particularly in agri-food supply chain management where perishable products create highly complex nondeterministic polynomial time (NP-hard) optimization problems (Chen et al., 2015; Sahebjamnia et al., 2018; Chouhan et al., 2021), i.e., it involves the optimization of multiple variables and may include multi-objective programming in which the number of possible combinations grow exponentially.

Additional promising applications include solving large-scale linear equation systems in animal breeding for estimating genetic merits across millions of animals, quantum-enhanced ML approaches for satellite image classification in land-use analysis, and quantum search algorithms for genome assembly to efficiently piece together DNA segments. Climate modeling is critical to on-farm decision-making and could also see improvements through QC’s ability to process multiple variables simultaneously; however, significant challenges remain, including understanding climate, defining sustainability metrics, and the nascent state of QC technology. Applications in the applied sciences, specifically agricultural sciences, including animal sciences, are vast and promising, but they are still theoretical or in the early stages of development, with most practical applications not expected in the near future due to current quantum hardware limitations(Tedeschi, 2024; Pook et al., 2025).

### Animal science

#### Animal nutrition and welfare

In animal nutrition, QC could revolutionize our ability to model complex biological systems by simulating quantum-level interactions among microbial communities, dietary compounds, and host tissues. In rumen microbiome analysis, QC could enable the exploration of dynamic interactions among thousands of microbial species, fermentation products, and host genetic responses, i.e., factors that influence nutrient utilization, microbial crossfeeding behavior, microbial protein synthesis, and methane emissions. For instance, the enzyme nitrogenase, crucial for microbial protein synthesis in the rumen, contains iron-molybdenum cofactors where electron transfer occurs through quantum tunneling (Hoffman et al., 2014). Current metagenomic and metabolomic studies produce terabytes of data that require extensive classical processing (Li et al., 2019). Quantum algorithms, such as quantum search and simulation methods, could reduce processing times and help identify microbial consortia optimized for feed efficiency and reduced emissions (Pook et al., 2025). These tools may also facilitate the screening of novel feed additives or compounds by modeling their effects on microbial metabolism and host responses, supporting more precise and sustainable nutrition strategies. Specifically, QC could model how methane inhibitors, such as 3-nitrooxypropanol (or other halogenated haloforms), interact with methyl-coenzyme M reductase at the quantum level, potentially identifying more effective inhibitor designs. Furthermore, quantum algorithms for solving partial differential equations have demonstrated potential in climate and environmental modeling, which could inform real-time heat stress predictions and guide the design of ventilation systems in livestock housing (Pook et al., 2025). Quantum principal component analysis has also shown promise in identifying key environmental and physiological features related to thermal comfort and facility design in livestock operations (Maraveas et al., 2024; Pook et al., 2025). This could optimize the interaction of multiple variables, including the temperature-humidity index, air velocity, radiant heat load, and animal-specific factors such as body weight and production level (Tedeschi and Fox, 2020a, b). Together, these applications illustrate how QC may enhance both the nutritional and environmental dimensions of precision livestock systems.

#### Animal breeding and genomics

In animal breeding specifically, QC has been proposed as a potential tool for certain computational challenges, though significant practical limitations remain. Current mixed model equations used in genetic evaluation require solving systems with billions of equations, as breeding programs now routinely evaluate millions of animals across multiple traits simultaneously (Vandenplas et al., 2023; Pook et al., 2025). The computational complexity of these systems has become a major bottleneck, with some evaluations requiring days of processing on high-performance computing clusters (Freudenberg et al., 2023). The Harrow-Hassidim-Lloyd quantum algorithm could theoretically provide speedups for solving certain linear systems, with runtime that is polynomial in log(N) and the condition number κ, provided the coefficient matrix is sparse and well-conditioned (Harrow et al., 2009). However, this approach is limited to estimating specific properties of the solution (such as expectation values) rather than computing individual breeding values directly. To address such limitations, Mukherjee and Basu Mallik (2025) recently introduced a hybrid quantum-classical algorithm designed to solve linear systems with reduced quantum resource demands, offering a potentially more practical alternative to Harrow-Hassidim-Lloyd for scientific computing problems requiring full solution vectors. Similarly, quantum-inspired classical algorithms have shown promise for low-rank linear systems with logarithmic dependence on dimension, though they still have polynomial dependence on other parameters like rank and condition number (Chia et al., 2020). Given that breeding value estimation typically requires the full solution vector and often involves poorly conditioned systems, the practical applicability of these quantum approaches to real breeding programs remains an open question, requiring further research (Pook et al., 2025). The applications of QC in livestock genomics extend beyond traditional sequence analysis to encompass the integration of complex multi-omics data. Grover’s algorithm provides quadratic speedup for genome assembly, reducing search complexity from O(N) to O(√N) for assembling fragmented DNA sequences (Sarkar et al., 2021; Fang et al., 2024). This quadratic speedup—where a classical search through N fragments requires N operations while Grover’s algorithm needs only √N operations—means that assembling a genome from 1 million fragments could theoretically require just 1,000 quantum operations instead of 1 million classical ones. This advancement is particularly relevant for livestock breeding programs where de novo assembly of reference genomes for indigenous breeds remains computationally challenging (Crysnanto et al., 2021), as also emphasized by Pook et al. (2025) in their assessment of QC applications in agriculture.

#### Precision livestock farming

The recent review by Maraveas et al. (2024) expands the discussion of QC in agriculture, specifically highlighting how QC, when combined with AI, could transform precision livestock farming (**PLF**). The transition to “smart agriculture” necessitates data-centric approaches, where the strategic integration of QC and AI offers unprecedented capabilities to enhance predictive analytics, optimize resource allocation, and model complex biological systems under real-world constraints (Maraveas et al., 2024; Tedeschi, 2024). A particularly promising area is QML, a rapidly advancing field at the intersection of QC and classical ML. The QML field leverages quantum-enhanced algorithms such as quantum neural networks, quantum kernel methods, and hybrid quantum–classical models to process high-dimensional datasets common in PLF. These include real-time sensor data on animal behavior, rumen fermentation dynamics, feed composition variability, and environmental conditions (Neethirajan, 2020; Tedeschi et al., 2021). Research in QML has also explored quantum generalizations of established ML models like Boltzmann machines, generative adversarial networks, and autoencoders (Allcock and Zhang, 2019), which may offer computational advantages for non-linear and complex agricultural datasets. Such approaches could support advanced pattern recognition, predictive modeling, and adaptive learning in data-intensive livestock systems. QML has the potential to enhance decision support systems that integrate mechanistic modeling with AI-driven inference—referred to as hybrid intelligent mechanistic models (Tedeschi, 2022; 2023). These systems may identify optimal feed interventions, mitigate emissions, or predict welfare risks under fluctuating conditions. While these quantum-enhanced methods remain largely conceptual, they offer a novel computational layer beyond what is currently achievable with classical AI, and represent a key frontier as quantum technology matures. In parallel, quantum optimization algorithms show promise for solving logistical and operational challenges in livestock systems. For instance, the QAOA could be applied to optimize feed delivery routes across multiple barns, reducing labor costs and ensuring timely feeding (Farhi et al., 2014). In integrated crop-livestock systems, quantum optimization could balance nutrient flows between crop and animal units, maximizing whole-farm resource efficiency while minimizing environmental impacts (Pook et al., 2025). Additionally, quantum digital twins represent an emerging and transformative application in PLF. These high-fidelity virtual replicas can simulate entire livestock production systems by integrating genomic, nutritional, health, environmental, and economic variables. Such models offer the potential for real-time optimization of animal management strategies, supporting individualized care and enhanced resource use. As described by Neethirajan and Kemp (2021), digital twins could enable predictive monitoring of animal health and welfare, leading to more responsive and sustainable farming practices. Maraveas et al. (2024) further suggest that quantum-enhanced simulations could significantly improve the fidelity and scalability of such digital twin systems. Together, these quantum technologies, QML, optimization, and digital twins, signal a paradigm shift in PLF, enabling systems that are more predictive, adaptive, and efficient than those relying solely on CC.

In summary, the value proposition of QC in agriculture ultimately depends on whether the field’s most pressing challenges involve quantum mechanical processes that classical computers fundamentally cannot simulate efficiently or optimization problems of such complexity that quantum algorithms would provide substantial speedups compared to classical approaches.

### Future considerations

Beyond the technical capabilities, we must also consider the social and economic implications of adopting QC in agriculture. The trajectory mirrors the early evolution of CC, when mainframes were exclusively accessible to military installations, research laboratories, and large corporations (Ceruzzi, 2003) due to their enormous cost and specialized maintenance requirements. Similarly, the high costs of quantum infrastructure may limit access to wealthy institutions and corporations, potentially widening the digital divide between large industrial farming operations and small-scale farmers. Questions of data ownership, algorithmic transparency, and the concentration of technological power become increasingly relevant. Additionally, the carbon footprint of QC facilities should be weighed against their potential environmental benefits in agricultural applications. Current QC systems, particularly those using superconducting qubits, require cryogenic cooling to ∼10–15 mK and complex control electronics that consume significant amounts of energy (Arute et al., 2019), that end up favoring CC for handling simple problems (Desdentado et al., 2024). While computational benefits for certain problem classes may be substantial, it is essential to consider whether these energy demands could offset environmental gains achieved through improved predictions or optimizations. Future QC adoption in agriculture will need to weigh computational benefits against energy costs to ensure net sustainability gains. A further consideration is the growing demand for robust quantum software and benchmarking tools to support reliable, scalable applications. System-level software is critical to translate quantum algorithms into executable instructions while managing control, error correction, and noise mitigation, which are tasks that remain computationally intensive and largely reliant on classical preprocessing. Importantly, software and hardware must evolve in unison. The effectiveness of QC hinges on tight integration between software capabilities and the physical constraints and architecture of quantum hardware. Without this co-development, advances in one domain may be bottlenecked by limitations in the other. Finally, a critical challenge is education: preparing the next generation of agricultural scientists with the interdisciplinary knowledge spanning quantum physics, computer science, and agricultural systems necessary to develop and implement quantum solutions. This educational transformation will require significant revisions to existing curricula and the development of specialized training programs that bridge the gap between quantum theory and practical agricultural applications.

## Conclusion

Quantum computing is not simply a faster version of CC, as it represents a new computational paradigm with the potential to solve classes of problems that remain out of reach for even the most advanced classical systems. Its promise lies in how it redefines problem-solving, especially for applications involving quantum mechanical systems, massive optimization spaces, or probabilistic modeling. Despite current limitations in hardware scalability, error correction, and coherence times, the theoretical and algorithmic groundwork continues to progress rapidly, laying the foundation for transformative applications once suitable quantum hardware becomes viable.

For agricultural sciences, including animal science, the potential of QC is both intriguing and largely speculative. Many of its proposed benefits remain largely theoretical, such as modeling rumen microbial interactions at the molecular level, optimizing feed formulations under dynamic environmental constraints, or integrating high-dimensional sensor data in real-time. Yet, the trajectory mirrors early CC: conceptual exploration preceding hardware readiness. A critical consideration remains whether QC truly offers solutions to fundamental agricultural problems that classical systems, including supercomputers and AI, cannot resolve. This question must guide research priorities as we evaluate potential quantum applications against existing classical approaches. Hybrid approaches, including quantum-enhanced AI, quantum simulators, and quantum-classical workflows, offer more immediate entry points into agricultural applications, especially for PLF and sustainability. We believe that QC is not positioned to replace CC but to complement it. Classical methods will remain the backbone for most agricultural systems, but QC may augment their capabilities in specific domains such as complex system modeling, resource optimization, and molecular simulation.

To prepare, applied science communities must begin building quantum readiness. This includes investing in quantum literacy among researchers, fostering interdisciplinary collaborations, identifying meaningful agricultural use cases, and engaging with quantum hardware and software ecosystem developers. Moreover, we must proactively address the social and economic implications of QC adoption in agriculture. The high costs of quantum infrastructure may limit access to wealthy institutions and corporations, potentially exacerbating the digital divide between large industrial farming operations and small-scale farmers. Questions of data ownership, algorithmic transparency, and the concentration of technological power become increasingly relevant and must be addressed through thoughtful policy development and inclusive stakeholder engagement. As we stand at the intersection of theory and implementation, the agricultural sciences have a rare opportunity to shape the trajectory of QC applications from the outset. The road ahead is long, but the potential returns, both scientifically and economically, as well as environmentally, make this journey well worth the investment.

## Supplementary Material

skaf445_Supplementary_Data

## Abbreviations

AI =: artificial intelligence
AWS =: Amazon Web Services
BCG =: Boston Consulting Group
CC =: classical computing
ENIAC =: electronic numerical integrator and computer
FTQC =: fault-tolerant quantum computer
ML =: machine learning
NASEM =: National Academies of Sciences, Engineering, and Medicine
NISQ =: noisy intermediate-scale quantum
PLF =: precision livestock farming
QAOA =: quantum approximate optimization algorithm
QC =: quantum computing
QML =: quantum machine learning
TRS =: Tandy Radio Shack
UNIVAC =: universal automatic computer

## References

[skaf445-B1] Aaronson S. 2015. Read the fine print. Nat. Phys. 11(4):291–293. 10.1038/nphys3272

[skaf445-B2] Acharya R. , Abanin D. A., Aghababaie-Beni L., Aleiner I., Andersen T. I., Ansmann M., Arute F., Arya K., Asfaw A., Astrakhantsev N. et al. 2025. Quantum error correction below the surface code threshold. Nature. 638(8052):920–926. 10.1038/s41586-024-08449-y 39653125 PMC11864966

[skaf445-B3] Aleksandrowicz , Alexander G. T., Barkoutsos P., Bello L., Ben-Haim Y., Bucher D., Cabrera-Hernández F. J., Carballo-Franquis J., Chen A., Chen C.-F. et al. 2019. Qiskit: An open-source framework for quantum computing. *Zenodo*. 10.5281/zenodo.2562111

[skaf445-B4] Allcock J , Zhang S. 2019. Quantum machine learning. Natl. Sci. Rev. 6(1):26–28. 10.1093/nsr/nwy149 34691828 PMC8291413

[skaf445-B5] Anonymous. 2019. A precarious milestone for quantum computing. Nature. 574:453–454. 10.1038/d41586-019-03168-1 31645738

[skaf445-B6] Anonymous. 2024a. NISQ versus FTQC in the 2025–2029 timeframe. Quantum Computing Report. [accessed April 20, 2025]. Available from https://quantumcomputingreport.com/nisq-versus-ftqc-in-the-2025-2029-timeframe/.

[skaf445-B7] Anonymous. 2024b. Quantinuum unveils accelerated roadmap to achieve universal, fully fault-tolerant quantum computing by 2030. Quantinuum. [accessed April 20, 2025]. Available from https://www.quantinuum.com/press-releases/quantinuum-unveils-accelerated-roadmap-to-achieve-universal-fault-tolerant-quantum-computing-by-2030

[skaf445-B8] Anonymous. 2025a. Amazon Web Services announces a new quantum computing chip. Amazon News. [accessed April 20, 2025]. Available from https://www.aboutamazon.com/news/aws/quantum-computing-aws-ocelot-chip

[skaf445-B9] Anonymous. 2025b. IBM reveals more details about its quantum error correction roadmap. Quantum Computing Report. [accessed August 9, 2025]. Available from https://quantumcomputingreport.com/ibm-reveals-more-details-about-its-quantum-error-correction-roadmap/

[skaf445-B10] Arute F. , Arya K., Babbush R., Bacon D., Bardin J. C., Barends R., Biswas R., Boixo S., Brandao F. G. S. L., Buell D. A. et al. 2019. Quantum supremacy using a programmable superconducting processor. Nature. 574(7779):505–510. 10.1038/s41586-019-1666-5 31645734

[skaf445-B11] Aspect A. 2023. The second quantum revolution: From basic concepts to quantum technologies. Pages 7-30 in Photonic Quantum Technologies. M. Benyoucef, ed. [accessed May 4, 2025]. Available from 10.1002/9783527837427.ch2 10.1002/9783527837427.ch2

[skaf445-B12] Aspect A. 2024. Einstein and the quantum revolutions. Chicago (IL): University of Chicago Press.

[skaf445-B13] Aspect A. , Grangier P., Roger G. 1982. Experimental realization of Einstein-Podolsky-Rosen-Bohm *gedankenexperiment*: a new violation of bell’s inequalities. Phys. Rev. Lett. 49(2):91–94. 10.1103/PhysRevLett.49.91

[skaf445-B14] Aspuru-Guzik A. , Dutoi A. D., Love P. J., Head-Gordon M. 2005. Simulated quantum computation of molecular energies. Science. 309(5741):1704–1707. 10.1126/science.1113479 16151006

[skaf445-B15] Aspuru-Guzik A. , Lindh R., Reiher M. 2018. The matter simulation (r)evolution. ACS Cent. Sci. 4(2):144–152. 10.1021/acscentsci.7b00550 29532014 PMC5832995

[skaf445-B16] Baiardi A. , Christandl M., Reiher M. 2023. Quantum computing for molecular biology. Chembiochem. 24(13):e202300120. 10.1002/cbic.202300120 37151197

[skaf445-B17] Baker B. 2024. Quantinuum unveils 2030 roadmap for fault-tolerant quantum computing. IOT World Today. [accessed April 20, 2025]. Available from https://www.iotworldtoday.com/quantum/quantinuum-unveils-2030-roadmap-for-fault-tolerant-quantum-computing

[skaf445-B18] Bell J. S. 1964. On the einstein podolsky rosen paradox. Physics Physique Fizika. 1(3):195–200. 10.1103/PhysicsPhysiqueFizika.1.195

[skaf445-B19] Bennett C. H. , DiVincenzo D. P. 2000. Quantum information and computation. Nature. 404(6775):247–255. 10.1038/35005001 10749200

[skaf445-B20] Bharti K. , Cervera-Lierta A., Kyaw T. H., Haug T., Alperin-Lea S., Anand A., Degroote M., Heimonen H., Kottmann J. S., Menke T. et al. 2022. Noisy intermediate-scale quantum algorithms. Rev. Mod. Phys. 94(1):015004. 10.1103/RevModPhys.94.015004

[skaf445-B21] Biamonte J. , Wittek P., Pancotti N., Rebentrost P., Wiebe N., Lloyd S. 2017. Quantum machine learning. Nature. 549(7671):195–202. 10.1038/nature23474 28905917

[skaf445-B22] Black , Kuhn P. E. D. R., Williams C. J. 2002. Quantum computing and communication. In: Advances in computers. vol. 56. Zelkowitz M. V., ed. Elsevier. p. 189–244. Available from https://www.sciencedirect.com/science/article/pii/S0065245802800079. 10.1016/S0065-2458(02)80007-9

[skaf445-B23] Blatner D. 2012. Spectrums: Our mind-boggling universe from infinitesimal to infinity. New York (NY): Walker & Co.

[skaf445-B24] Blekos K. , Brand D., Ceschini A., Chou C.-H., Li R.-H., Pandya K., Summer A. 2024. A review on quantum approximate optimization algorithm and its variants. Phys. Rep. 1068:1–66. 10.1016/j.physrep.2024.03.002

[skaf445-B25] Bobier , Langione J.-F. M., Naudet-Baulieu C., Cui Z., Watanabe E. 2024. The long-term forecast for quantum computing still looks bright. Boston Consulting Group. [accessed April 20, 2025]. ­Available from https://www.bcg.com/publications/2024/long-term-forecast-for-quantum-computing-still-looks-bright

[skaf445-B26] Bobier , Langione J.-F. M., Tao E., Gourevitch A. 2021. What happens when ‘if’ turns to ‘when’ in quantum computing? 16p. [accessed April 18, 2025]. Available from https://web-assets.bcg.com/89/00/d2d074424a6ca820b1238e24ccc0/bcg-what-happens-when-if-turns-to-when-in-quantum-computing-jul-2021-r.pdf

[skaf445-B27] Born M , Einstein A. 1971. The Born-Einstein letters; correspondence between albert einstein and max and hedwig born from 1916 to 1955. London (UK): Macmillan Press.

[skaf445-B28] Braunstein S. L. , van Loock P. 2005. Quantum information with continuous variables. Rev. Mod. Phys. 77(2):513–577. 10.1103/RevModPhys.77.513

[skaf445-B29] Burnett J. J. , Bengtsson A., Scigliuzzo M., Niepce D., Kudra M., Delsing P., Bylander J. 2019. Decoherence benchmarking of superconducting qubits. NPJ Quantum Inf. 5(1):54. 10.1038/s41534-019-0168-5

[skaf445-B30] Camino B. , Buckeridge J., Warburton P. A., Kendon V., Woodley S. M. 2023. Quantum computing and materials science: a practical guide to applying quantum annealing to the configurational analysis of materials. J. Appl. Phys. 133(22):221102. 10.1063/5.0151346

[skaf445-B31] Campbell E. 2024. A series of fast-paced advances in quantum error correction. Nat. Rev. Phys. 6(3):160–161. 10.1038/s42254-024-00706-3

[skaf445-B32] Cao Y. , Romero J., Olson J. P., Degroote M., Johnson P. D., Kieferová M., Kivlichan I. D., Menke T., Peropadre B., Sawaya N. P. D. et al. 2019. Quantum chemistry in the age of quantum computing. Chem. Rev. 119(19):10856–10915. 10.1021/acs.chemrev.8b00803 31469277

[skaf445-B33] Cerezo M. , Verdon G., Huang H.-Y., Cincio L., Coles P. J. 2022. Challenges and opportunities in quantum machine learning. Nat. Comput. Sci. 2(9):567–576. 10.1038/s43588-022-00311-3 38177473

[skaf445-B34] Ceruzzi P. E. 2003. A history of modern computing. 2nd ed. Cambridge (MA): MIT Press.

[skaf445-B35] Chen Y. T. , Chan F. T. S., Chung S. H. 2015. An integrated closed-loop supply chain model with location allocation problem and product recycling decisions. Int. J. Prod. Res. 53(10):3120–3140. 10.1080/00207543.2014.975849

[skaf445-B36] Cherney M. A. 2025. Google says commercial quantum computing applications arriving within five years. Reuters. [accessed April 20, 2025]. Available from https://www.reuters.com/technology/google-says-commercial-quantum-computing-applications-arriving-within-five-years-2025-02-05/

[skaf445-B37] Chia N. , Gilyén A., Lin H., Lloyd S., Tang E., Wang C. 2020. Quantum-inspired algorithms for solving low-rank linear equation systems with logarithmic dependence on the dimension. Leibniz Int. Proc. Inform. LIPIcs. 181(47):1–17. 10.4230/LIPIcs.ISAAC.2020.47

[skaf445-B38] Chouhan V. K. , Khan S. H., Hajiaghaei-Keshteli M. 2021. Metaheuristic approaches to design and address multi-echelon sugarcane closed-loop supply chain network. Soft Comput. 25(16):11377–11404. 10.1007/s00500-021-05943-7

[skaf445-B39] Clarke J , Wilhelm F. K. 2008. Superconducting quantum bits. Nature. 453(7198):1031–1042. 10.1038/nature07128 18563154

[skaf445-B40] Collins H , Nay C. 2022. IBM unveils 400 qubit-plus quantum processor and next-generation IBM quantum system two. IBM Newsroom. [accessed April 20, 2025]. Available from https://newsroom.ibm.com/2022-11-09-IBM-Unveils-400-Qubit-Plus-Quantum-Processor-and-Next-Generation-IBM-Quantum-System-Two

[skaf445-B41] Cross , Bishop A. W. L. S., Smolin J. A., Gambetta J. M. 2017. Open quantum assembly language. *arXiv*.1707.03429. 10.48550/arXiv.1707.03429, preprint: not peer reviewed.

[skaf445-B42] Crysnanto D. , Leonard A. S., Fang Z.-H., Pausch H. 2021. Novel functional sequences uncovered through a bovine multiassembly graph. Proc. Natl. Acad. Sci. U S A. 118(20):e2101056118. 10.1073/pnas.2101056118 33972446 PMC8157972

[skaf445-B43] Das A , Chakrabarti B. K. 2008. Colloquium: quantum annealing and analog quantum computation. Rev. Mod. Phys. 80(3):1061–1081. 10.1103/RevModPhys.80.1061

[skaf445-B44] de Souza K. X. S. , Bolfe É. L., Leite M. A. D. A., Bambini M. D., Visoli M. C., Júnior A. L., da Silva F. R., Esquerdo J. C. D. M., Carvalho J. E. D. 2024. Quantum computing: current and potential applications in digital agriculture. Pesq. Agropec. Bras. 59:e03753. 10.1590/S1678-3921.pab2024.v59.03753

[skaf445-B45] Desdentado E. , Calero C., Moraga M. Á., Serrano M., García F. 2024. Exploring the trade-off between computational power and energy efficiency: an analysis of the evolution of quantum computing and its relation to classical computing. J. Syst. Softw. 217:112165. 10.1016/j.jss.2024.112165

[skaf445-B46] Deutsch D. 1985. Quantum theory, the Church–Turing principle and the universal quantum computer. Proc. R. Soc. Lond. A. Math. Phys. Sci. 400(1818):97–117. 10.1098/rspa.1985.0070

[skaf445-B47] Dyakonov M. I. 2020. Will We ever have a quantum computer? Cham: Springer. 10.1007/978-3-030-42019-2

[skaf445-B48] Einstein A. , Podolsky B., Rosen N. 1935. Can quantum-mechanical description of physical reality be considered complete? Phys. Rev. 47(10):777–780. 10.1103/PhysRev.47.777

[skaf445-B49] Fang J.-K. , Lin Y.-F., Huang J.-H., Chen Y., Fan G.-M., Sun Y., Feng G., Guo C., Meng T., Zhang Y. et al. 2024. Divide-and-conquer quantum algorithm for hybrid *de novo* genome assembly of short and long reads. PRX Life. 2(2):023006. 10.1103/PRXLife.2.023006

[skaf445-B50] Farhi , Goldstone E. J., Gutmann S. 2014. A quantum approximate optimization algorithm. *arXiv*.1411.4028. 10.48550/arXiv.1411.4028, preprint: not peer reviewed.

[skaf445-B51] Feynman R. P. 1982. Simulating physics with computers. Int. J. Theor. Phys. 21(6–7):467–488. 10.1007/BF02650179

[skaf445-B52] Fowler A. G. , Mariantoni M., Martinis J. M., Cleland A. N. 2012. Surface codes: towards practical large-scale quantum computation. Phys. Rev. A. 86(3):032324. 10.1103/PhysRevA.86.032324

[skaf445-B53] Freudenberg , Schlather A. M., Vandenplas J., Pook T. 2023. Accelerating single-step evaluations through GPU offloading. In: Proceedings of the 2023 Interbull Meeting, v. 59. Lyon, France; p. 23–32. [accessed May 31, 2025]. Available from https://journal.interbull.org/index.php/ib/article/view/1893

[skaf445-B54] Georgescu I. M. , Ashhab S., Nori F. 2014. Quantum simulation. Rev. Mod. Phys. 86(1):153–185. 10.1103/RevModPhys.86.153

[skaf445-B55] Griffiths D. J. , Schroeter D. F. 2018. Introduction to quantum mechanics. 3rd ed. Cambridge (UK): Cambridge University Press. 10.1017/9781316995433

[skaf445-B56] Grover L. K. 1996. A fast quantum mechanical algorithm for database search. In: Proceedings of the twenty-eighth annual ACM symposium on Theory of Computing, Philadelphia, Pennsylvania, USA. Association for Computing Machinery; p. 212–219. 10.1145/237814.237866

[skaf445-B57] Harrigan M. P. , Sung K. J., Neeley M., Satzinger K. J., Arute F., Arya K., Atalaya J., Bardin J. C., Barends R., Boixo S. et al. 2021. Quantum approximate optimization of non-planar graph problems on a planar superconducting processor. Nat. Phys. 17(3):332–336. 10.1038/s41567-020-01105-y

[skaf445-B58] Harrow A. W. , Hassidim A., Lloyd S. 2009. Quantum algorithm for linear systems of equations. Phys. Rev. Lett. 103(15):150502. 10.1103/PhysRevLett.103.150502 19905613

[skaf445-B59] Harrow A. W. , Montanaro A. 2017. Quantum computational supremacy. Nature. 549(7671):203–209. 10.1038/nature23458 28905912

[skaf445-B60] Hauke P. , Katzgraber H. G., Lechner W., Nishimori H., Oliver W. D. 2020. Perspectives of quantum annealing: methods and implementations. Rep. Prog. Phys. 83(5):054401. 10.1088/1361-6633/ab85b8 32235066

[skaf445-B61] Hoffman B. M. , Lukoyanov D., Yang Z.-Y., Dean D. R., Seefeldt L. C. 2014. Mechanism of nitrogen fixation by nitrogenase: the next stage. Chem. Rev. 114(8):4041–4062. 10.1021/cr400641x 24467365 PMC4012840

[skaf445-B62] Horodecki R. , Horodecki P., Horodecki M., Horodecki K. 2009. Quantum entanglement. Rev. Mod. Phys. 81(2):865–942. 10.1103/RevModPhys.81.865

[skaf445-B63] Hossenfelder S. 2022. Existential physics; a scientist’s guide to life’s biggest questions. New York (NY): Viking.

[skaf445-B64] Ivezic M. 2024. IBM unveils 156-qubit ‘Heron R2’ quantum processor. PostQuantum. [accessed August 9, 2025]. Available from https://postquantum.com/industry-news/ibm-heron-r2-quantum/

[skaf445-B65] Gangapuram , A. J., A. Läuchli, and C. Hempel. 2024. Benchmarking quantum computer simulation software packages: State vector simulators. SciPost Phys. Core. 7(4):075. 10.21468/SciPostPhysCore.7.4.075

[skaf445-B66] Kaiser S , Granade C. 2021. Learn quantum computing with Python and Q#; a hands-on approach. Shelter Island (NY): Manning Publications. Available from https://livebook.manning.com/book/learn-quantum-computing-with-python-and-q-sharp

[skaf445-B67] Kim Y. , Eddins A., Anand S., Wei K. X., van den Berg E., Rosenblatt S., Nayfeh H., Wu Y., Zaletel M., Temme K. et al. 2023. Evidence for the utility of quantum computing before fault tolerance. Nature. 618(7965):500–505. 10.1038/s41586-023-06096-3 37316724 PMC10266970

[skaf445-B68] Krantz P. , Kjaergaard M., Yan F., Orlando T. P., Gustavsson S., Oliver W. D. 2019. A quantum engineer’s guide to superconducting qubits. Appl. Phys. Rev. 6(2):021318. 10.1063/1.5089550

[skaf445-B69] Li Z. , Gao N., Martini J. W. R., Simianer H. 2019. Integrating gene expression data into genomic prediction. Front. Genet. 10:126. 10.3389/fgene.2019.00126 30858865 PMC6397893

[skaf445-B70] Maraveas C. , Konar D., Michopoulos D. K., Arvanitis K. G., Peppas K. P. 2024. Harnessing quantum computing for smart agriculture: empowering sustainable crop management and yield optimization. Comput. Electron. Agric. 218:108680. 10.1016/j.compag.2024.108680

[skaf445-B71] McArdle S. , Endo S., Aspuru-Guzik A., Benjamin S. C., Yuan X. 2020. Quantum computational chemistry. Rev. Mod. Phys. 92(1):015003. 10.1103/RevModPhys.92.015003

[skaf445-B72] Mermin N. D. 1981. Quantum mysteries for anyone. J. Philos. 78(7):397–408. 10.2307/2026482

[skaf445-B73] Mermin N. D. 2007. Quantum computer science: an introduction. Cambridge (MA): Cambridge University Press. Available from https://www.cambridge.org/core/product/66462590D10C8010017CF1D7C45708D7. 10.1017/CBO9780511813870

[skaf445-B74] Moll N. , Barkoutsos P., Bishop L. S., Chow J. M., Cross A., Egger D. J., Filipp S., Fuhrer A., Gambetta J. M., Ganzhorn M. et al. 2018. Quantum optimization using variational algorithms on near-term quantum devices. Quantum Sci. Technol. 3(3):030503. 10.1088/2058-9565/aab822

[skaf445-B75] Montanaro A. 2016. Quantum algorithms: an overview. NPJ Quantum Inf. 2(1):15023. 10.1038/npjqi.2015.23 PMC12137133 40487024

[skaf445-B76] Moore G. E. 1965. Cramming more components onto integrated circuits. Electronics. (Basel). 38(8):114–117.

[skaf445-B77] Mukherjee A , Basu Mallik B. 2025. Transforming precision agriculture with quantum computing: a novel algorithm for boosting crop yields and optimizing resources. EPJ Web Conf. 325(:01004.

[skaf445-B78] National Academies of Sciences, Engineering, and Medicine. 2019. Quantum computing: progress and prospects. Washington (DC): National Academies Press. 10.17226/25196

[skaf445-B79] Neethirajan S. 2020. The role of sensors, big data and machine learning in modern animal farming. Sens. Biosensing. Res. 29:100367. 10.1016/j.sbsr.2020.100367

[skaf445-B80] Neethirajan S , Kemp B. 2021. Digital twins in livestock farming. Animals (Basel). 11(4). 10.3390/ani11041008 PMC8065673 33916713

[skaf445-B81] Nielsen M. A. , Chuang I. L. 2010. Quantum computation and quantum information: 10th anniversary edition. Cambridge (MA): Cambridge University Press. Available from https://www.cambridge.org/core/product/01E10196D0A682A6AEFFEA52D53BE9AE. 10.1017/CBO9780511976667

[skaf445-B82] Noiri A. , Takeda K., Nakajima T., Kobayashi T., Sammak A., Scappucci G., Tarucha S. 2022. Fast universal quantum gate above the fault-tolerance threshold in silicon. Nature. 601(7893):338–342. 10.1038/s41586-021-04182-y 35046603

[skaf445-B83] Novet J. 2025. Amazon touts its first quantum computing chip a week after Microsoft’s unveiling. CNBC. [accessed April 20, 2025]. Available from https://www.cnbc.com/2025/02/27/amazon-touts-its-first-quantum-computing-chip-a-week-after-microsofts-unveiling.html

[skaf445-B84] Pasternack A. 2024. IBM built the biggest, coolest quantum computer. Now comes the hard part. FastCompany. [accessed May 31, 2025]. Available from https://www.fastcompany.com/90992708/ibm-quantum-system-two

[skaf445-B85] Pednault , Gunnels E. J. A., Nannicini G., Horesh L., Wisnieff R. 2019. Leveraging secondary storage to simulate deep 54-qubit Sycamore circuits. *arXiv*.1910.09534. 10.48550/arXiv.1910.09534, preprint: not peer reviewed.

[skaf445-B86] Pook T. , Vandenplas J., Boschero J. C., Aguilera E., Leijnse K., Chauhan A., Bouzembrak Y., Knapen R., Aldridge M. 2025. Assessing the potential of quantum computing in agriculture. Comput. Electron. Agric. 235:110332. 10.1016/j.compag.2025.110332

[skaf445-B87] Preskill J. 2018. Quantum computing in the NISQ era and beyond. Quantum. 2:79. 10.22331/q-2018-08-06-79

[skaf445-B88] Preskill J. 2023. Crossing the quantum chasm: From NISQ to fault tolerance. Quantum Frontiers. [accessed April 20, 2025]. Available from https://quantumfrontiers.com/2023/12/09/crossing-the-quantum-chasm-from-nisq-to-fault-tolerance/

[skaf445-B89] Roberson T. M. , White A. G. 2019. Charting the Australian quantum landscape. Quantum Sci. Technol. 4(2):020505. 10.1088/2058-9565/ab02b4

[skaf445-B90] Rozenman G. G. , Fu S., Arie A., Shemer L. 2019. Quantum mechanical and optical analogies in surface gravity water waves. In Fluids. 4(2):96. 10.3390/fluids4020096

[skaf445-B91] Saffman M. 2019. Quantum computing with neutral atoms. Natl. Sci. Rev. 6(1):24–25. 10.1093/nsr/nwy088 34691827 PMC8291449

[skaf445-B92] Saffman M. , Walker T. G., Mølmer K. 2010. Quantum information with Rydberg atoms. Rev. Mod. Phys. 82(3):2313–2363. 10.1103/RevModPhys.82.2313

[skaf445-B93] Sahebjamnia N. , Fathollahi-Fard A. M., Hajiaghaei-Keshteli M. 2018. Sustainable tire closed-loop supply chain network design: hybrid metaheuristic algorithms for large-scale networks. J. Clean. Prod. 196:273–296. 10.1016/j.jclepro.2018.05.245

[skaf445-B94] Sarkar A. , Al-Ars Z., Bertels K. 2021. Quaser: Quantum accelerated *de novo* DNA sequence reconstruction. PLoS One. 16(4):e0249850. 10.1371/journal.pone.0249850 33844699 PMC8041170

[skaf445-B95] Schlosshauer M. , Kofler J., Zeilinger A. 2013. A snapshot of foundational attitudes toward quantum mechanics. Stud Hist Philos Sci Part B Stud Hist Philos Modern Phys. 44(3):222–230. 10.1016/j.shpsb.2013.04.004

[skaf445-B96] Schuld M , Petruccione F. 2021. Machine learning with quantum computers. Cham: Springer. 10.1007/978-3-030-83098-4

[skaf445-B97] Shor P. 1994. Algorithms for quantum computation: discrete logarithms and factoring. In: 2013 IEEE 54th Annual Symposium on Foundations of Computer Science. Santa Fe, NM, USA: IEEE; p. 124–134. 10.1109/SFCS.1994.365700

[skaf445-B98] Shor P. W. 1997. Polynomial-time algorithms for prime factorization and discrete logarithms on a quantum computer. SIAM J. Comput. 26(5):1484–1509. 10.1137/S0097539795293172

[skaf445-B99] Skosana U , Tame M. 2021. Demonstration of Shor’s factoring algorithm for N = 21 on IBM quantum processors. Sci. Rep. 11(1):16599. 10.1038/s41598-021-95973-w 34400695 PMC8368060

[skaf445-B100] Stackpole B. 2024. Quantum computing: what leaders need to know now. MIT Management Sloan Scholl. [accessed April 20, 2025]. Available from https://mitsloan.mit.edu/ideas-made-to-matter/quantum-computing-what-leaders-need-to-know-now

[skaf445-B101] Tatananni M. 2025. IBM unveils plans for first ‘fault-tolerant’ quantum supercomputer. Barron’s. [accessed August 9, 2025]. Available from https://www.barrons.com/articles/ibm-quantum-supercomputer-af375f6e

[skaf445-B102] Tedeschi L. O. 2019. ASN-ASAS SYMPOSIUM: FUTURE OF DATA ANALYTICS IN NUTRITION: Mathematical modeling in ruminant nutrition: approaches and paradigms, extant models, and thoughts for upcoming predictive analytics. J. Anim. Sci. 97(5):1921–1944. 10.1093/jas/skz092 30882142 PMC6488328

[skaf445-B103] Tedeschi L. O. 2022. ASAS-NANP SYMPOSIUM: MATHEMATICAL MODELING IN ANIMAL NUTRITION: the progression of data analytics and artificial intelligence in support of sustainable development in animal science. J. Anim. Sci. 100(6):1–11. 10.1093/jas/skac111 PMC9171329 35412610

[skaf445-B104] Tedeschi L. O. 2023. Review: the prevailing mathematical modelling classifications and paradigms to support the advancement of sustainable animal production. Animal. 17 Suppl 5:100813. 10.1016/j.animal.2023.100813 37169649

[skaf445-B105] Tedeschi L. O. 2024. 6 Transforming animal agriculture through hybrid modeling and quantum computing. J. Anim. Sci. 102(Supplement_3):70–71. 10.1093/jas/skae234.078

[skaf445-B106] Tedeschi L. O. , Fox D. G. 2020a. The ruminant nutrition system: an applied model for predicting nutrient requirements and feed utilization in ruminants. Vol. 1. (3rd ed.) (3rd repr.). Dubuque (IA): Kendall Hunt. First published in 2020 by XanEdu, Ann Arbor, MI. [accessed February 7, 2025]. Available from https://he.kendallhunt.com/product/ruminant-nutrition-system-vol-1

[skaf445-B107] Tedeschi L. O. , Fox D. G. 2020b. The ruminant nutrition system: tables of equations and coding. Vol. 2. (3rd repr.). Dubuque (IA): Kendall Hunt. First published in 2020 by XanEdu, Ann Arbor, MI. [accessed February 7, 2025]. Available from https://he.kendallhunt.com/product/ruminant-nutrition-system-vol-2

[skaf445-B108] Tedeschi L. O. , Greenwood P. L., Halachmi I. 2021. Advancements in sensor technology and decision support intelligent tools to assist smart livestock farming. J. Anim. Sci. 99(2):1–11. 10.1093/jas/skab038 PMC7896629 33550395

[skaf445-B109] Terhal B. M. 2015. Quantum error correction for quantum memories. Rev. Mod. Phys. 87(2):307–346. 10.1103/RevModPhys.87.307

[skaf445-B110] Turing A. M. 1937. On computable numbers, with an application to the Entscheidungsproblem. Proc. Lond. Math. Soc. s2-42(1):230–265. https://doi.org/10.1112/plms/s2-42.1.230

[skaf445-B111] Vandenplas J. , ten Napel J., Darbaghshahi S. N., Evans R., Calus M. P. L., Veerkamp R., Cromie A., Mäntysaari E. A., Strandén I. 2023. Efficient large-scale single-step evaluations and indirect genomic prediction of genotyped selection candidates. Genet. Sel. Evol. 55(1):37. 10.1186/s12711-023-00808-z 37291510 PMC10251624

[skaf445-B112] Waldrop M. M. 2016. The chips are down for Moore’s law. Nature. 530(7589):144–147. 10.1038/530144a 26863965

[skaf445-B113] Wang D. S. , Fowler A. G., Hollenberg L. C. L. 2011. Surface code quantum computing with error rates over 1%. Phys. Rev. A. 83(2):020302. 10.1103/PhysRevA.83.020302

[skaf445-B114] Welsh D , Welsh T. 2007. Priming the Pump: How TRS-80 Enthusiasts Helped Spark the PC Revolution. The Seeker Books, Ferndale, MI

[skaf445-B115] Willsch D. , Willsch M., Jin F., De Raedt H., Michielsen K. 2023. Large-scale simulation of Shor’s quantum factoring algorithm. In Mathematics. 11(19):4222. 10.3390/math11194222

[skaf445-B116] Yin J. , Cao Y., Li Y.-H., Liao S.-K., Zhang L., Ren J.-G., Cai W.-Q., Liu W.-Y., Li B., Dai H. et al. 2017. Satellite-based entanglement distribution over 1200 kilometers. Science. 356(6343):1140–1144. 10.1126/science.aan3211 28619937

[skaf445-B117] Young , Scese K. M., Ebnenasir A. 2023. Simulating quantum computations on classical machines: a survey. *arXiv*.2311.16505. 10.48550/arXiv.2311.16505, preprint: not peer reviewed.

[skaf445-B118] Yung M.-H. 2019. Quantum supremacy: some fundamental concepts. Natl. Sci. Rev. 6(1):22–23. 10.1093/nsr/nwy072 34691826 PMC8291594

[skaf445-B119] Zhong H.-S. , Wang H., Deng Y.-H., Chen M.-C., Peng L.-C., Luo Y.-H., Qin J., Wu D., Ding X., Hu Y. et al. 2020. Quantum computational advantage using photons. Science. 370(6523):1460–1463. 10.1126/science.abe8770 33273064

[skaf445-B120] Zhou , Stoudenmire Y. E. M., Waintal X. 2020. What limits the simulation of quantum computers? Phys. Rev. X. 10(4):041038. 10.1103/PhysRevX.10.041038

[skaf445-B121] Zurek W. H. 2003. Decoherence, einselection, and the quantum origins of the classical. Rev. Mod. Phys. 75(3):715–775. 10.1103/RevModPhys.75.715

