# Supplementary material for: ASAS-NANP SYMPOSIUM: MATHEMATICAL MODELING IN ANIMAL NUTRITION: quantum computing in agricultural sciences: from theory to reality
Source: J Anim Sci. 2025 Dec 18;104:skaf445. doi: 10.1093/jas/skaf445 (PMC12930474; doi:10.1093/jas/skaf445)
Supplement: skaf445_Supplementary_Data [file skaf445_supplementary_data.docx]

**Appendix 1**. Classical versus quantum calculation approaches.

**Classical Approach**

Two calculations and a comparison are needed: 1. compute f(0), 2. compute f(1), and 3. compare: if f(0) = f(1), the function is constant; otherwise, it’s balanced.

**Quantum approach via Deutsch’s algorithm**

The quantum mechanics and algorithm for these calculations are discussed by Deutsch (1985), Nielsen and Chuang (2010), Mermin (2007), and Deutsch and Jozsa (1992), and implemented in Qiskit (Harkins, 2022). Deutsch’s algorithm represents the first quantum algorithm that definitively proves a quantum computer can solve a problem with fewer operations than any possible classical algorithm (Deutsch, 1985). It was later generalized to the Deutsch-Jozsa algorithm, which handles functions with multiple input bits. This, in turn, influenced the development of more powerful quantum algorithms, such as Shor’s factoring algorithm and Grover’s search algorithm (Jozsa, 1998). The $|0\rangle and |1\rangle$ are called “ket” notations (from Dirac’s bra-ket notation) (Nielsen and Chuang, 2010) and represent the two possible states of a qubit:

|0⟩ is the quantum equivalent of the classical bit 0

|1⟩ is the quantum equivalent of the classical bit 1

**Qubit**: Unlike classical bits that must be either 0 or 1, qubits can exist in a superposition of both states simultaneously, represented as $a|0\rangle+ b|1\rangle$, where a and b are complex numbers satisfying $|a|^{2} + |b|^{2} = 1$. A qubit has a two-dimensional state space in which |0⟩ and |1⟩ form an orthonormal basis, as follows:

$|0\rangle=\left[ \begin{matrix} 1 \\ 0 \end{matrix} \right]$, and $|1\rangle=\left[ \begin{matrix} 0 \\ 1 \end{matrix} \right]$

**1. Initial state**

We start with two qubits initialized as:

$$|\psi₀\rangle=|0\rangle\otimes|1\rangle$$

Where ⊗ represents the tensor product, meaning we have a system of two qubits, with the first in state |0⟩ and the second in state |1⟩.

Assuming this canonical basis, the Kronecker product of the vectors yields:

$$\left| \psi₀ \right\rangle=\left[ \begin{matrix} 1 \\ 0 \end{matrix} \right]\otimes\left[ \begin{matrix} 0 \\ 1 \end{matrix} \right]=\left[ \begin{matrix} 1\times\left[ \begin{matrix} 0 \\ 1 \end{matrix} \right] \\ 0\times\left[ \begin{matrix} 0 \\ 1 \end{matrix} \right] \end{matrix} \right]=\left[ \begin{matrix} \begin{matrix} 0 \\ 1 \end{matrix} \\ \begin{matrix} 0 \\ 0 \end{matrix} \end{matrix} \right]$$

**2. Hadamard gate (H)**

The Hadamard gates create superposition, allowing us to evaluate the function on all possible inputs simultaneously. The first qubit enters superposition $(|0\rangle+|1\rangle)/\surd2$, representing both possible inputs (0 and 1) simultaneously. The second qubit is prepared in the state $(|0\rangle-|1\rangle)/\surd2$, which is crucial for phase kickback. This is the key to quantum parallelism. When applying the Hadamard gate (H) transforms:

$$H=\frac{1}{\sqrt{2}}\left[ \begin{matrix} 1 & 1 \\ 1 & -1 \end{matrix} \right]$$

To both qubits, we obtain:

$H\left| 0 \right\rangle=\frac{1}{\sqrt{2}}\left[ \begin{matrix} 1 & 1 \\ 1 & -1 \end{matrix} \right]\left[ \begin{matrix} 1 \\ 0 \end{matrix} \right]= \frac{1}{\sqrt{2}}\left[ \begin{matrix} 1 \\ 1 \end{matrix} \right]=(|0\rangle+|1\rangle)/\surd2$, which is known as $|+\rangle$

$H\left| 1 \right\rangle=\frac{1}{\sqrt{2}}\left[ \begin{matrix} 1 & 1 \\ 1 & -1 \end{matrix} \right]\left[ \begin{matrix} 0 \\ 1 \end{matrix} \right]= \frac{1}{\sqrt{2}}\left[ \begin{matrix} 1 \\ -1 \end{matrix} \right]=(|0\rangle-|1\rangle)/\surd2$, which is known as $|-\rangle$

Thus, performing the tensor product, we obtain:

$$|\psi₁\rangle=H|0\rangle\otimes H|1\rangle$$

$$|\psi₁\rangle=(|0\rangle+ |1\rangle)/\surd2 \otimes(|0\rangle- |1\rangle)/\surd2$$

$$|\psi₁\rangle=(|00\rangle- |01\rangle+ |10\rangle- |11\rangle)/2$$

Where |xy⟩ is shorthand for |x⟩ ⊗ |y⟩.

Or, assuming,

$$\left| 00 \right\rangle=\left[ \begin{matrix} \begin{matrix} 1 \\ 0 \end{matrix} \\ \begin{matrix} 0 \\ 0 \end{matrix} \end{matrix} \right], \left| 01 \right\rangle=\left[ \begin{matrix} \begin{matrix} 0 \\ 1 \end{matrix} \\ \begin{matrix} 0 \\ 0 \end{matrix} \end{matrix} \right], \left| 10 \right\rangle=\left[ \begin{matrix} \begin{matrix} 0 \\ 0 \end{matrix} \\ \begin{matrix} 1 \\ 0 \end{matrix} \end{matrix} \right], and \left| 11 \right\rangle=\left[ \begin{matrix} \begin{matrix} 0 \\ 0 \end{matrix} \\ \begin{matrix} 0 \\ 1 \end{matrix} \end{matrix} \right]$$

We obtain:

$$\left| \psi₁ \right\rangle=\frac{1}{2}\left( \left[ \begin{matrix} \begin{matrix} 1 \\ 0 \end{matrix} \\ \begin{matrix} 0 \\ 0 \end{matrix} \end{matrix} \right]-\left[ \begin{matrix} \begin{matrix} 0 \\ 1 \end{matrix} \\ \begin{matrix} 0 \\ 0 \end{matrix} \end{matrix} \right]+\left[ \begin{matrix} \begin{matrix} 0 \\ 0 \end{matrix} \\ \begin{matrix} 1 \\ 0 \end{matrix} \end{matrix} \right]-\left[ \begin{matrix} \begin{matrix} 0 \\ 0 \end{matrix} \\ \begin{matrix} 0 \\ 1 \end{matrix} \end{matrix} \right] \right)= \left[ \begin{matrix} \begin{matrix} \frac{1}{2} \\ -\frac{1}{2} \end{matrix} \\ \begin{matrix} \frac{1}{2} \\ -\frac{1}{2} \end{matrix} \end{matrix} \right]$$

This creates a superposition where all possible input-output combinations have equal amplitude as their sum of squares is 1.

**3. Oracle Function**

When we need to evaluate a function in a way that preserves quantum superposition, the oracle encodes the function’s output in the phase of the quantum state rather than directly measuring it. This relies on a technique called “phase kickback,” as follows: when the second qubit is in the state $(|0\rangle-|1\rangle)/\surd2$, the function’s result gets encoded as a phase on the first qubit. For constant functions, the first qubit’s state becomes $(|0\rangle+|1\rangle)/\surd2$ (same phase). For balanced functions, the first qubit’s state becomes $(|0\rangle-|1\rangle)/\surd2$ (opposite phase). The quantum oracle Uₑ is defined as:

$$Uₑ|x\rangle|y\rangle= |x\rangle|y \oplus f(x)\rangle$$

Where ⊕ represents bitwise *xor* (i.e., exclusive *or*). That means if the bits are the same, *xor* returns zero; otherwise, *xor* returns 1.

Starting with our state after Hadamard gates:

$$|\psi₁\rangle= (|00\rangle- |01\rangle+ |10\rangle- |11\rangle)/2$$

Applying Uₑ to this state:

$$|\psi₂\rangle=Uₑ|\psi₁\rangle$$

$$|\psi₂\rangle=Uₑ[(|00\rangle- |01\rangle+ |10\rangle- |11\rangle)/2]$$

$$|\psi₂\rangle=(|0\rangle|0 \oplus f(0)\rangle- |0\rangle|1 \oplus f(0)\rangle+ |1\rangle|0 \oplus f(1)\rangle- |1\rangle|1 \oplus f(1)\rangle)/2$$

Now, let’s analyze what happens for each type of function:

**For constant functions:**

**Case 1**: If f(0) = f(1) = 0:

$$|\psi₂\rangle=(|00\rangle- |01\rangle+ |10\rangle- |11\rangle)/2$$

To factorize this, we group terms by the second qubit:

$$|\psi₂\rangle=(|00\rangle+ |10\rangle)/2 - (|01\rangle+ |11\rangle)/2$$

$$|\psi₂\rangle=(|0\rangle+ |1\rangle)/\surd2 \otimes|0\rangle/\surd2 - (|0\rangle+ |1\rangle)/\surd2 \otimes|1\rangle/\surd2$$

$$|\psi₂\rangle=(|0\rangle+ |1\rangle)/\surd2 \otimes(|0\rangle- |1\rangle)/\surd2$$

**Case 2**: If f(0) = f(1) = 1:

$$|\psi₂\rangle=(|01\rangle- |00\rangle+ |11\rangle- |10\rangle)/2$$

$$|\psi₂\rangle=(-|00\rangle+|01\rangle-|10\rangle+|11\rangle)/2$$

$$|\psi₂\rangle=-(|00\rangle-|01\rangle+|10\rangle-|11\rangle)/2$$

Following the same grouping:

$$|\psi₂\rangle=-[(|00\rangle+ |10\rangle)/2 - (|01\rangle+ |11\rangle)/2]$$

$$|\psi₂\rangle=-(|0\rangle+ |1\rangle)/\surd2 \otimes(|0\rangle- |1\rangle)/\surd2$$

$$|\psi₂\rangle=(|0\rangle+ |1\rangle)/\surd2 \otimes(|0\rangle- |1\rangle)/\surd2$$

**For balanced functions:**

**Case 1:** If f(0) = 0, f(1) = 1:

$$|\psi₂\rangle=(|00\rangle- \left| 01 \right\rangle+ |11\rangle-|10\rangle)/2$$

Rearranging to group by second qubit:

$$|\psi₂\rangle=(|00\rangle- |10\rangle)/2 + (|11\rangle- |01\rangle)/2$$

$$|\psi₂\rangle=(|0\rangle- |1\rangle)/\surd2 \otimes|0\rangle/\surd2 + (|1\rangle- |0\rangle)/\surd2 \otimes|1\rangle/\surd2$$

$$|\psi₂\rangle=(|0\rangle- |1\rangle)/\surd2 \otimes|0\rangle/\surd2 - (|0\rangle- |1\rangle)/\surd2 \otimes|1\rangle/\surd2$$

$$|\psi₂\rangle=(|0\rangle- |1\rangle)/\surd2 \otimes(|0\rangle- |1\rangle)/\surd2$$

**Case 2:** If f(0) = 1, f(1) = 0:

$$|\psi₂\rangle=(|01\rangle- \left| 00 \right\rangle+ \left| 10 \right\rangle- |11\rangle)/2$$

Rearranging:

$$|\psi₂\rangle=-(|00\rangle- |01\rangle- |10\rangle+ |11\rangle)/2$$

$$|\psi₂\rangle=-[(|00\rangle- |10\rangle)/2 - (|01\rangle- |11\rangle)/2]$$

$$|\psi₂\rangle=-[(|0\rangle- |1\rangle)/\surd2 \otimes|0\rangle/\surd2 - (|0\rangle- |1\rangle)/\surd2 \otimes|1\rangle/\surd2]$$

$$|\psi₂\rangle=-(|0\rangle- |1\rangle)/\surd2 \otimes(|0\rangle- |1\rangle)/\surd2$$

$$|\psi₂\rangle=(|0\rangle- |1\rangle)/\surd2 \otimes(|0\rangle- |1\rangle)/\surd2$$

This can be rewritten as:

$$|\psi₂\rangle= \pm(|0\rangle\pm|1\rangle)/\surd2\otimes(|0\rangle-|1\rangle)/\surd2$$

Where the first ± is + for constant functions and – for balanced functions.

1. **Apply Final Hadamard to First Qubit**

The information about the function is now encoded in the phase relationship of the first qubit, but quantum phases cannot be directly measured. The Hadamard transforms this phase information into amplitude information, as follows:

$H(|0\rangle+|1\rangle)/\surd2 = |0\rangle$ (for constant functions)

$H(|0\rangle-|1\rangle)/\surd2 = |1\rangle$ (for balanced functions)

This converts the invisible phase information into a measurable difference in the computational basis. Applying H to the first qubit, we obtain:

$$|\psi₃\rangle=H\otimes I|\psi₂\rangle$$

For constant functions, this becomes:

$$|\psi₃\rangle=|0\rangle\otimes(|0\rangle-|1\rangle)/\surd2$$

For balanced functions, this becomes:

$$|\psi₃\rangle=|1\rangle\otimes(|0\rangle-|1\rangle)/\surd2$$

**5. Measurement**

Measuring the first qubit: If we get |0⟩, the function is constant. If we get |1⟩, the function is balanced.

**References**

Deutsch, D. 1985. Quantum theory, the Church–Turing principle and the universal quantum computer. *Proceedings of the Royal Society of London. A. Mathematical and Physical Sciences*. 400 (1818):97-117. doi: 10.1098/rspa.1985.0070

Deutsch, D., and R. Jozsa. 1992. Rapid solution of problems by quantum computation. *Proceedings of the Royal Society of London. Series A: Mathematical and Physical Sciences*. 439 (1907):553-558. doi: 10.1098/rspa.1992.0167

Harkins, F. 2022. Deutsch-Jozsa algorithm. Available: <https://github.com/Qiskit/textbook/blob/main/notebooks/ch-algorithms/deutsch-jozsa.ipynb>.

Jozsa, R. 1998. Quantum algorithms and the Fourier transform. *Proceedings of the Royal Society of London. Series A: Mathematical, Physical and Engineering Sciences*. 454 (1969):323-337. doi: 10.1098/rspa.1998.0163

Mermin, N. D. 2007. Quantum Computer Science: An Introduction. Cambridge University Press, Cambridge, MA. Available at: <https://www.cambridge.org/core/product/66462590D10C8010017CF1D7C45708D7>. doi: 10.1017/CBO9780511813870

Nielsen, M. A., and I. L. Chuang. 2010. Quantum Computation and Quantum Information: 10th Anniversary Edition. Cambridge University Press, Cambridge, MA. Available at: <https://www.cambridge.org/core/product/01E10196D0A682A6AEFFEA52D53BE9AE>. doi: 10.1017/CBO9780511976667
